# Transformation and gene editing in the bioenergy grass *Miscanthus*

**DOI:** 10.1186/s13068-022-02241-8

**Published:** 2022-12-28

**Authors:** Anthony Trieu, Mohammad B. Belaffif, Pradeepa Hirannaiah, Shilpa Manjunatha, Rebekah Wood, Yokshitha Bathula, Rebecca L. Billingsley, Anjali Arpan, Erik J. Sacks, Thomas E. Clemente, Stephen P. Moose, Nancy A. Reichert, Kankshita Swaminathan

**Affiliations:** 1 HudsonAlpha Institute for Biotechnology, 601 Genome Way, Huntsville, AL 35806 USA; 2 Department of Biological Sciences, Mississippi State University, 295 Lee Blvd., Mississippi State, MS 39762 USA; 3 Department of Crop Sciences, E.R. Madigan Laboratory, University of Illinois Urbana-Champaign, 1201 W. Gregory Dr., Urbana, IL 61801 USA; 4 Department of Agronomy and Horticulture, University of Nebraska-Lincoln, Lincoln, NE 68583 USA; 5 DOE Center for Advanced Bioenergy and Bioproducts Innovation, University of Illinois Urbana-Champaign, Urbana, IL 61801 USA

**Keywords:** *Miscanthus sinensis*, *Miscanthus sacchariflorus*, *Miscanthus* x *giganteus*, Transformation, CRISPR/Cas9, Gene editing, *Lemon white1* (*lw1*)

## Abstract

**Background:**

*Miscanthus*, a C4 member of Poaceae, is a promising perennial crop for bioenergy, renewable bioproducts, and carbon sequestration. Species of interest include nothospecies *M.* x *giganteus* and its parental species *M. sacchariflorus* and *M. sinensis*. Use of biotechnology-based procedures to genetically improve *Miscanthus*, to date, have only included plant transformation procedures for introduction of exogenous genes into the host genome at random, non-targeted sites.

**Results:**

We developed gene editing procedures for *Miscanthus* using CRISPR/Cas9 that enabled the mutation of a specific (targeted) endogenous gene to knock out its function. Classified as paleo-allopolyploids (duplicated ancient *Sorghum*-like DNA plus chromosome fusion event), design of guide RNAs (gRNAs) for *Miscanthus* needed to target both homeologs and their alleles to account for functional redundancy. Prior research in *Zea mays* demonstrated that editing the *lemon white1* (*lw1*) gene, involved in chlorophyll and carotenoid biosynthesis, via CRISPR/Cas9 yielded pale green/yellow, striped or white leaf phenotypes making *lw1* a promising target for visual confirmation of editing in other species. Using sequence information from both *Miscanthus* and sorghum, orthologs of maize *lw1* were identified; a multi-step screening approach was used to select three gRNAs that could target homeologs of *lw1*. Embryogenic calli of *M. sacchariflorus*, *M. sinensis* and *M.* x *giganteus* were transformed via particle bombardment (biolistics) or *Agrobacterium tumefaciens* introducing the *Cas9* gene and three gRNAs to edit *lw1*. Leaves on edited *Miscanthus* plants displayed the same phenotypes noted in maize. Sanger sequencing confirmed editing; deletions in *lw1* ranged from 1 to 26 bp in length, and one deletion (433 bp) encompassed two target sites. Confocal microscopy verified lack of autofluorescence (chlorophyll) in edited leaves/sectors.

**Conclusions:**

We developed procedures for gene editing via CRISPR/Cas9 in *Miscanthus* and, to the best of our knowledge, are the first to do so. This included five genotypes representing three *Miscanthus* species. Designed gRNAs targeted all copies of *lw1* (homeologous copies and their alleles); results also confirmed *lw1* made a good editing target in species other than *Z. mays*. The ability to target specific loci to enable endogenous gene editing presents a new avenue for genetic improvement of this important biomass crop.

**Graphical Abstract:**

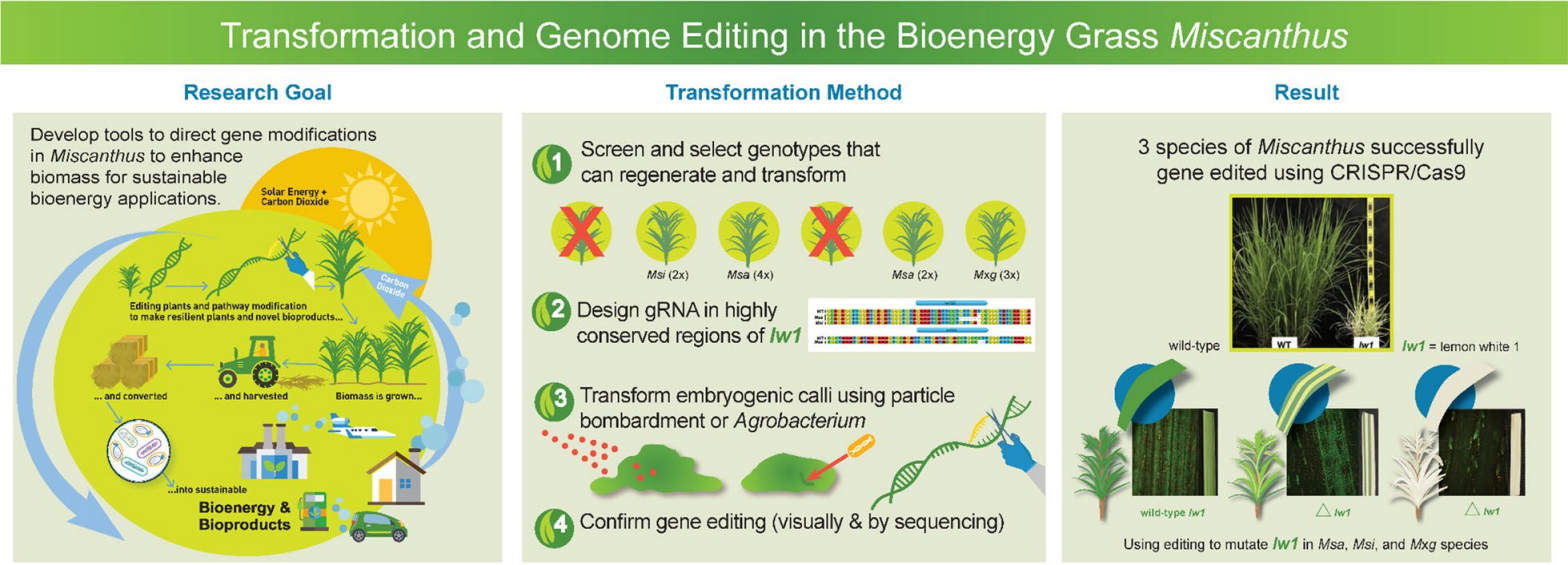

**Supplementary Information:**

The online version contains supplementary material available at 10.1186/s13068-022-02241-8.

## Background

The genus *Miscanthus* is a C4 member of the Poaceae (grass) family that has a basic chromosome number of 19 [1, 2]. The most important species of *Miscanthus* for growth as feedstocks for bioenergy and bioproducts is the hybrid (classified as nothospecies) *M.* x *giganteus* (*M*x*g*), and its parental species *M. sacchariflorus* (*Msa*) and *M. sinensis* (*Msi*) [3–5]. *Miscanthus* can be grown on marginal land, usually does not need external nitrogen or herbicide applications, has moderate-to-good drought tolerance, and efficiently stores nutrients in rhizomes and roots for the following growing season [6, 7]. In a 6-year study in the midwestern U.S. (in the States of Illinois, Iowa and Nebraska), *M*x*g* out-performed three other warm-season crops that included *Panicum virgatum* (switchgrass), *Andropogon* sp. (big bluestem) and *Bouteloua* sp. (grama grass) [8]. A global biomass yield dataset that analyzed hundreds of articles (data from 31 countries representing all continents except Antarctica) indicated that *Miscanthus* generated greater yields compared to switchgrass, *Populus* sp. (poplar) and *Salix* sp. (willow), and was second only to *Eucalyptus* sp. [9].

The genetics of individual *Miscanthus* species can be quite complex, with the number of basic chromosome sets ranging from two to six (2x–6x) [2]. Genome analyses revealed a close syntenic relationship between *Miscanthus* and *Sorghum bicolor*; *Msi* DNA appeared to be composed of ancient sorghum-like DNA that had been doubled, along with one chromosomal fusion event that occurred post-doubling, so the *Miscanthus* genus is composed of paleo-allopolyploids [10–12]. Due to diploidization that also occurred post-doubling, *Miscanthus* spp. have two homeologous chromosomes that are highly syntenic, so functional redundancy between the homeologous gene pairs is highly likely.

Based on yield alone, *Miscanthus* is a promising bioenergy crop, but could be enhanced through focused classical breeding as well as biotechnology approaches for introduction of exogenous genes or modification of existing endogenous genes. For these latter approaches, plant tissue culture-based regeneration and DNA introduction procedures are needed. *Miscanthus* has been successfully regenerated from immature inflorescences; the earliest reported success in *Msi* generated embryogenic calli on a medium containing the plant growth regulator 2,4-dichlorophenoxyacetic acid (2,4-D) then transferred to media for embryo maturation/regeneration [13]. Those researchers were also the first to determine that immature inflorescences, harvested 3–4 weeks prior to flowering, were the most responsive and yielded the greatest number of regenerants. Successes in other *Miscanthus* species followed using immature inflorescences, and included *M*x*g* although initially referred to as *Miscanthus* ‘Giganteus’ [14], and *Msa* [15, 16]. The focus of these and other *Miscanthus* procedures that involved regeneration from embryogenic calli was on the use of immature inflorescences as explants (reviewed by [17]). Since *Miscanthus* plants are self-incompatible, this precluded the use of seeds for clonal regeneration of the mother plant, and explant choices for *M*x*g* were limited due to its sterility.

Two species of *Miscanthus* have been successfully transformed using *Agrobacterium tumefaciens* or particle bombardment: *Msa* [18–20], and *Msi* [21–25]. Trieu [26] gave specific examples for transformation of *M*x*g* which is confirmed in this report.

Aside from traditional transformation for insertion of exogenous genes into a plant’s genome, gene editing can be used to mutate a targeted gene to knock out or reduce expression of that gene, and can also be used to add (knock in) DNA into a specific location in the targeted genome. The CRISPR/Cas9 system (clustered regularly interspaced short palindromic repeats/CRISPR-associated protein) has been developed into a powerful tool to precisely edit plant genomes. *Oryza sativa* (rice) was the first member of Poaceae to yield edited plants using CRISPR/Cas9 [27–30]. Since then, the CRISPR/Cas9 system has been used to modify endogenous genes for important agronomic traits in numerous plant species, including *Zea mays* (maize), rice, sorghum, *Hordeum* sp. (barley), *Saccharum* sp. (sugarcane), and *Triticum* sp. (wheat) (reviewed by [31–33]). To the best of our knowledge, there are no reports on gene editing in any *Miscanthus* species.

In multiple plant species, initial efforts to demonstrate that gene editing had taken place were in targeted genes where mutations produced easily visualized phenotypes. The phytoene desaturase gene (*pds*) was such a target in eudicots and monocots; tissues containing edited (knocked out) *pds* displayed a chlorotic/white morphology (reviewed by [31–33]). Unfortunately, use of *pds* as a target in *Miscanthus* led to detrimental effects on tissue growth and vigor (data not shown). A similar type of gene is *lemon white1* (*lw1*) identified in maize and located on the long arm of chromosome 1; it was named for the phenotype produced upon loss of function [34]. Homozygous mutant maize seeds displayed a lemon-colored endosperm compared to yellow in wild-type, and seedlings lacked chlorophyll and carotenoids [35]. Targeting the *lw1* gene in maize via CRISPR/Cas9 resulted in white leaves on shoots/plantlets [36, 37], but leaf phenotypes could range from white to pale green/yellow to striped [38]. An approach was devised that identified maize *lw1* orthologs in both *Miscanthus* and sorghum for development of gene editing procedures in *Miscanthus*.

This report demonstrates that the genomes of three *Miscanthus* species (*Msa*-diploid and tetraploid, *Msi*-diploid, and *M*x*g*-triploid) can be edited via the CRISPR/Cas9 system. This report also demonstrates the successful targeting of *lw1* for use as a visual marker for gene mutation (knockout) in species other than maize. The gRNAs designed to target homeologous *lw1* genes successfully mutated the *lw1* copies in *Miscanthus*. Given that *M*x*g* is the primary *Miscanthus* grown commercially for bioenergy and bioproducts, the ability to conduct targeted gene editing on it, as well as its parental species (*Msa* and *Msi*), will be highly valuable for future improvement of this crop. To enable gene editing, transformation and regeneration procedures were developed and optimized for *Miscanthus*.

## Results and discussion

### In vitro  responses—genotype screening, transformation efficiencies and paromomycin dose–response curve

Of the 87 *Miscanthus* genotypes screened for in vitro responses, the majority (51) were incapable of generating embryogenic calli on the media used in these screenings (CIM, RM; Additional file 1: Table S1). Among the 36 capable of generating embryogenic calli, 14 genotypes had responses rated as good or very good (41–80% of the immature inflorescences generated embryogenic calli). Additional file 1: Fig. S1A shows examples of embryogenic calli from 18 successful genotypes. Embryogenic calli from each of the 35 genotypes assessed for shoot regeneration could successfully generate shoots, and the majority of response ratings (83%) were categorized the same in both variables assessed (such as ability to generate embryogenic calli = good, and ability to regenerate shoots = good; Additional file 1: Table S1). Among the 13 genotypes assessed for transformation efficiency (Additional file 1: Table S1, Fig. S1B), some trends were noted. In general, transformation efficiencies were higher when biolistics was employed (range: 3.7–9%) compared to *A. tumefaciens* (range: 0–5.7%). The increased efficiency of biolistics varied among genotypes; *Msa* S1 had a 9% transformation efficiency with biolistics and 0% with *A. tumefaciens*, whereas the difference was not as great with *Msi* UI1 (3.7% with biolistics, 2.5% with *A. tumefaciens*).

When developing procedures for regeneration alone or coupled with transformation, it is important to determine the best genotypes to work with since responses can vary dramatically; in *Miscanthus*, responses ranged from 0 to 80% (Additional file 1: Table S1). The majority of genotypes did not respond on the media used, so if genotypes of interest were among the non-responders, media composition would have been the focus. Media chosen for use in screenings had been refined in our labs so the focus was on which genotypes responded the best knowing that we wanted representatives of *Msa*, *Msi* and *M*x*g* included in our gene editing experiments. The five genotypes with good culture responses and comparatively high shoot regeneration rates (Additional file 1: Table S1) selected for gene editing included *Msa* ‘Tohoku-2010-020’ (S1, tetraploid), *Msa* RU2012-110 (S13, diploid), *Msi* ‘Purpurascens’ (P1, diploid), *Msi* 10UI-008-2011-1-Row Replicated—CHA-115-7 (UI1, diploid), and *M*x*g* Freedom (triploid).

The concentration of paromomycin in callus maintenance medium (CMM-1; Table 1) affected callus growth of *Msi* UI1 wild-type calli (assessed after 3 weeks). Visible growth and calli weights decreased with increased concentrations of paromomycin (Additional file 1: Fig. S2). Based on results, paromomycin concentrations of 100–200 mg L^−1^ were used for selection of transgenic, gene edited *Miscanthus* tissues; concentrations used were species-dependent (Table 1).

**Table 1:** Additives to media used for culture initiation, transformation and plant regeneration in three *Miscanthus* species

Media	*Msa* & *Msi*	*M*x*g*
Callus induction media (CIM)	CIM-1: 2 mg 2,4-D + 0.25 mg zeatin	CIM-2: 3 mg 2,4-D + 0.1 mg BA^1^
Callus maintenance media (CMM)	CMM-1: 2 mg 2,4-D + 0.25 mg zeatin; 3.64 g mannitol and sorbitol	same as CIM-2
Callus osmotic medium (COM); biolistics	COM-1: CIM-1 with 36.4 g mannitol and sorbitol^2^	N/A
Coculture media (CCM); *A. tumefaciens*	CCM-1: 2 mg 2,4-D + 0.25 mg zeatin; 0.1X MS salts, 0.1X B5 vitamins, 30 g maltose, 10 g glucose, 100 mg L-cysteine, pH 5.4; 39.24 mg acetosyringone (200 μM)	CCM-2: CIM-2 plus 58.86 mg acetosyringone (300 μM)
Recovery media (RCM); *A. tumefaciens*	RCM-1: CIM-1 plus 100 mg timentin	RCM-2: CIM-2 plus 300 mg cefotaxime
Callus selection media (CSM)	CSM-1: CIM-1 with paromomycin (200 mg *Msac*, 100 mg *Msin*); CSM-1 T: recipe above plus 100 mg timentin	CSM-2: CIM-2 plus 300 mg cefotaxime and 100 mg paromomycin
Regeneration selection media (RSM); no selection = RM	RSM-1: 2 mg BA + 0.1 mg NAA; 20 g sucrose, 10 g maltose^3^; paromomycin (200 mg *Msac*, 100 mg *Msin*); RSM-1 T: recipe above plus 100 mg timentin	RSM-2: 5 mg BA + 0.24 mg NAA; 20 g sucrose, no MgCl_2_ or L-proline^1^; 300 mg cefotaxime and 50 mg paromomycin
Rooting selection media (RtSM); no selection = RtM	RtSM-1: 0.25 mg IBA; 0.5X MS salts, 1X B5 vitamins, 15 g sucrose, 0.1 mg myo-inositol; 100 mg paromomycin	RtM-2: 0.5X for MS salts, vitamins and sucrose; pH 5.8; no MgCl_2_ or L-proline

The media sequence when starting with immature inflorescences. Plant growth regulators and non-standard additives included with amounts given per liter; further details provided in text. *Msa* and *Msi* media designations followed by -1; *M*x*g* media followed by -2

*Msa* *M. sacchariflorus*, *Msi* *M. sinensis*, *M*x*g* *M. x giganteus*

^1^Reference [61]

^2^Sugar alcohols based on [19]

^3^Reference [26]

### Post-transformation responses—selection on antibiotics and confirmation of stable transformation

Transgenic shoots typically arose from embryogenic calli transformed via biolistics 1–2 weeks earlier than those transformed via *A. tumefaciens*. This was likely due to the presence of antibiotics in the latter tissue culture media (Table 1) to deter further growth of *A. tumefaciens* post-transformation. Addition of antibiotics for this purpose was noted to be detrimental to monocot plant tissues in culture [39, 40]. In general, *A. tumefaciens* transformed *Miscanthus* calli were often visibly darker (produced phenolics) compared to those that had been transformed via biolistics. Regardless of the type of transformation system used, transgenic plants were generated in all three *Miscanthus* species and leaf phenotypes indicated that *lw1* edits had taken place.

PCR was used to initially screen putative transgenic tissues for presence of the *neomycin phosphotransferase II* (*nptII*) gene delivered by pHA194 (Additional file 1: Fig. S3) to confirm transformation (Additional file 1: Fig. S4); PCR results further confirmed that any noted *lw1* mutant phenotypes were a result of editing and most likely not a result of spontaneous mutation. Since multiple shoots could be regenerated from an individual callus piece, each piece was numbered (event number; Additional file 1: Fig. S4) so shoots with different event numbers were independent transformants. Multiple shoots from an event were also given letter designations to go with the event number to indicate they might not be independent transformants.

### Phenotypic analysis of *lw1* edited plants

**Fig. 1:**
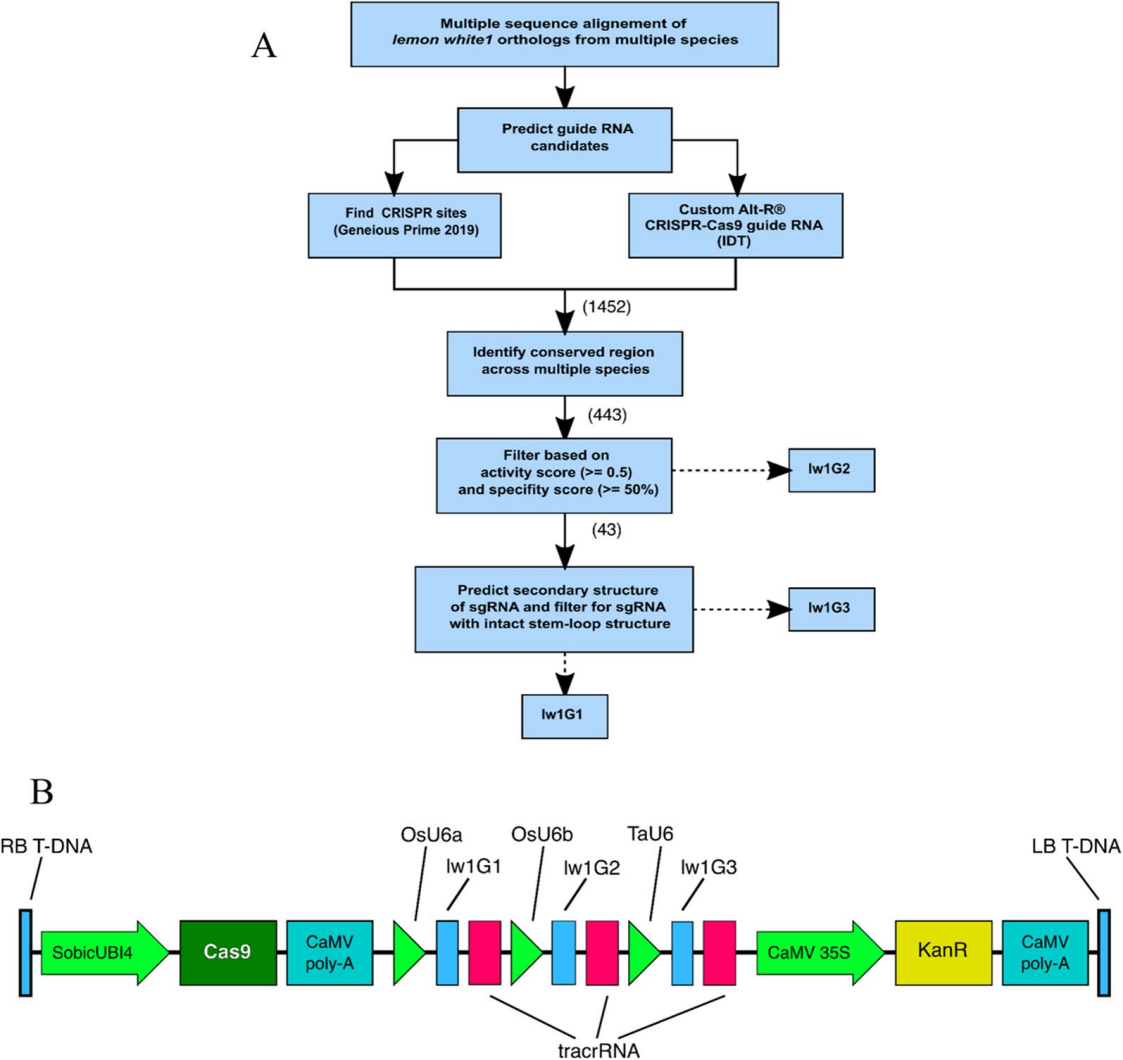
Design and implementation of CRISPR/Cas9 guide RNA for gene editing in *Miscanthus*. **A** Flowchart of the steps taken to identify gRNAs that target a gene of interest. The numbers in parentheses correspond to the number of gRNA candidates that passed the previous filtering criteria. Three gRNAs, lw1G1, lw1G2 and lw1G3, targeting the *lw1* gene in *Miscanthus* were selected based on the different filtering criteria in this flowchart. **B** Structure of the T-DNA cassette in plasmid pHA194, containing the *Cas9* gene, KanR (*nptII*) selectable marker, and the three gRNAs, each driven by a different U6 promoter

The gRNAs were designed to edit all copies of *lw1* using a multi-step pipeline (Fig. 1) that considered the evolutionary relationship between sorghum and *Miscanthus* in the design to ensure editing of homeologous and allelic copies of *lw1* in all genotypes. With the history of whole genome duplication within the genus *Miscanthus* compared to sorghum, a diploid *Miscanthus* will have at most four allelic copies of *lw1*, while triploid and tetraploid *Miscanthus* will have at most six and eight allelic copies, respectively (Additional file 1: Fig. S5).

**Fig. 2:**
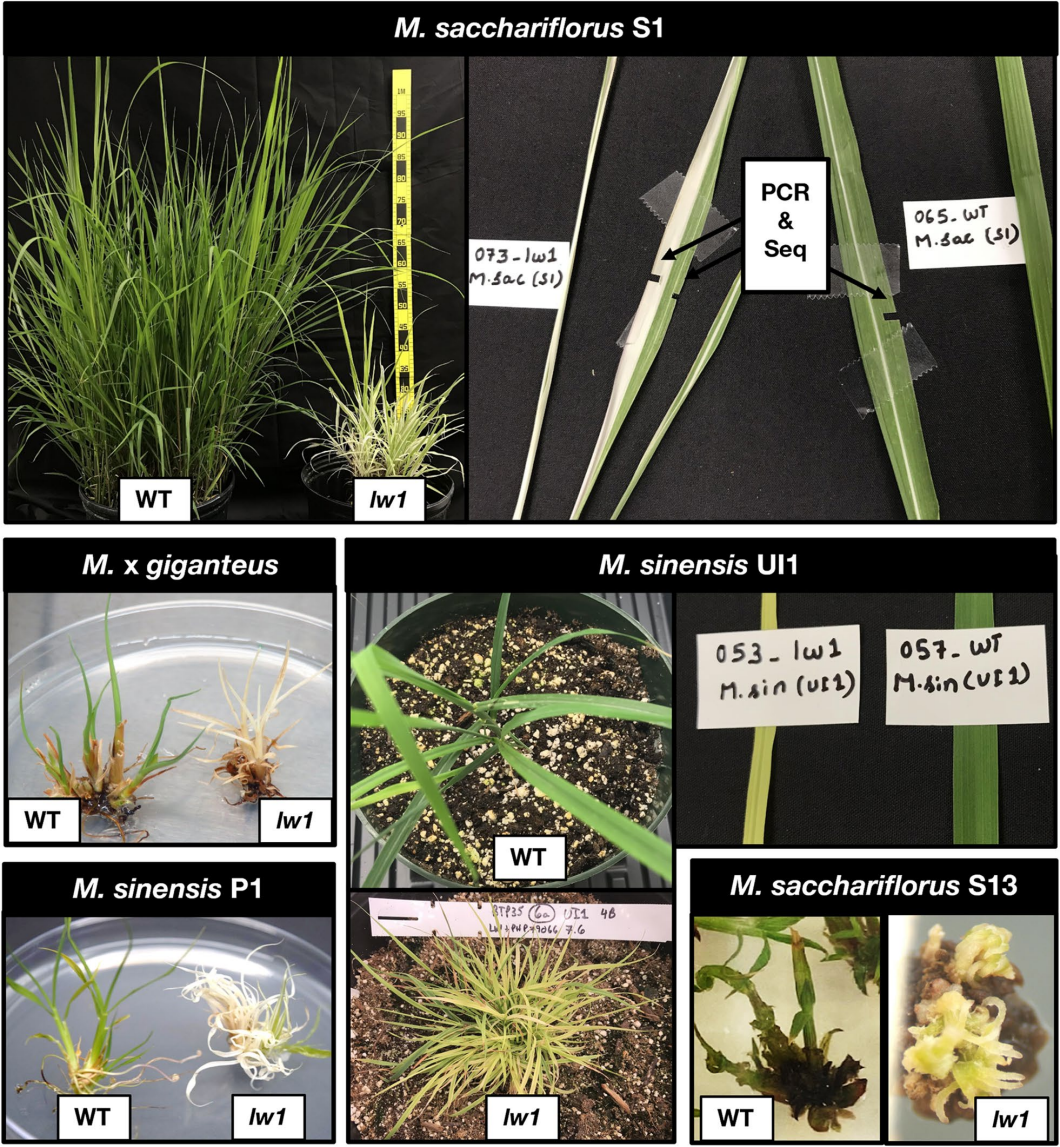
Leaf phenotypes in five *Miscanthus* genotypes transformed with gene editing vector pHA194. Examples of altered leaf colors (pale green/yellow, striped, white) noted in *Miscanthus* regenerants arising from embryogenic calli transformed with pHA194 (contained *Cas9* gene and three gRNA targeting *lw1*). Five genotypes are represented, two transformed via biolistics (*Msa* S1, *Msi* UI1) and three via *A. tumefaciens* (*Msa* S13, *Msi* P1, *M*x*g* Freedom). WT = isogenic wild-type line, *lw1* = transgenic edited shoots/plantlets/plants

Mutation/knockout of all *lw1* copies would result in a white phenotype in leaves. Both transformation methods produced *lw1* edited *Miscanthus* plants that exhibited a range of leaf phenotypes: pale green/yellow, striped (white or pale yellow), and solid white; these phenotypes mirrored leaf phenotypes noted in maize *lw1* CRISPR/Cas9 knockouts [37]. All five *Miscanthus* genotypes displayed leaves that were edited, and an example of each genotype is shown in Fig. 2. Similar phenotypes were observed in edited plants, irrespective of the species or genotype. It was difficult to keep the solid white mutants alive, and many of these were lost at the plantlet stage.

**Fig. 3:**
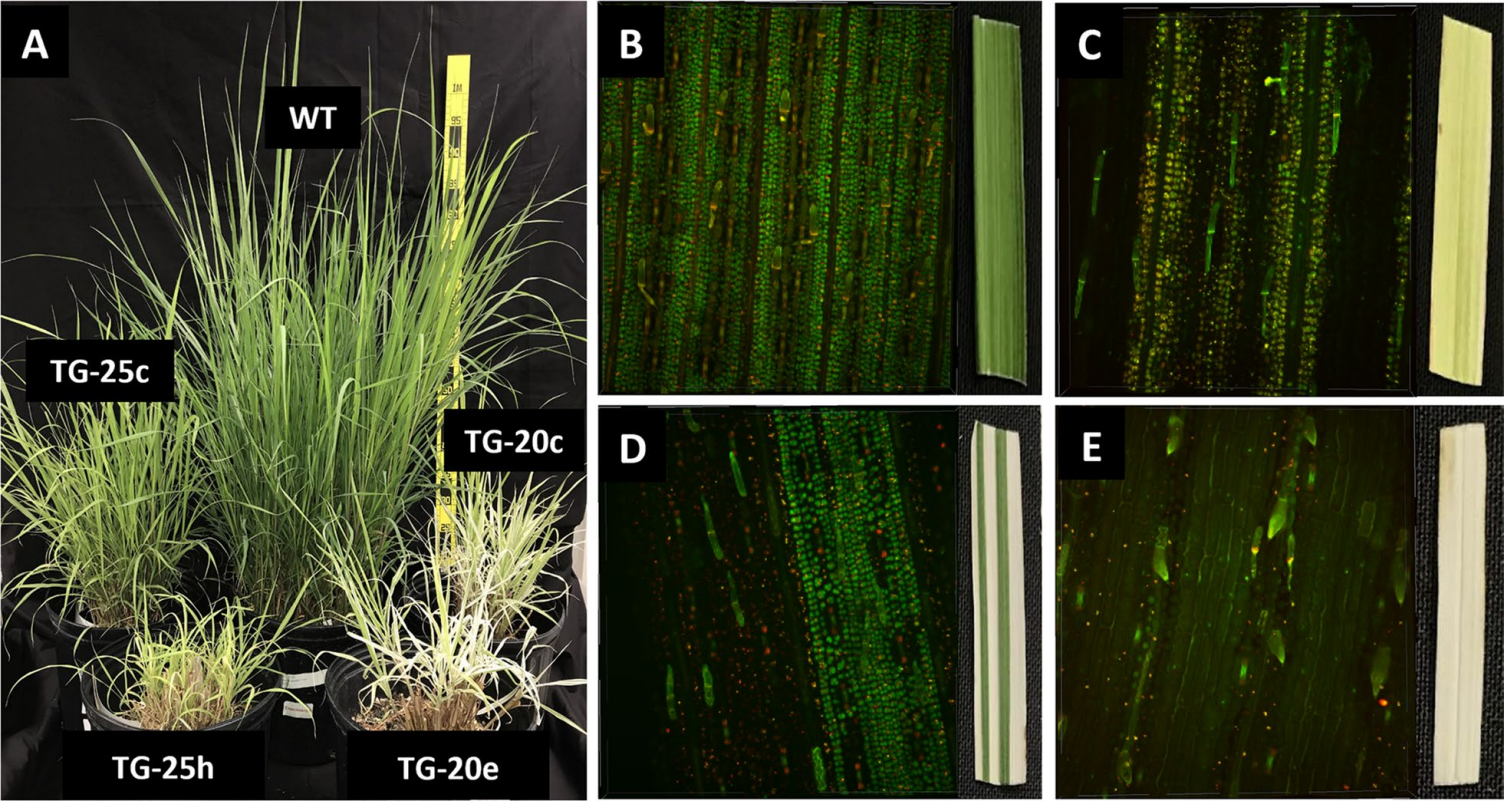
Confocal images of *M. sacchariflorus* S1 leaves from wild-type and TG-20 *lw1* edited plants. Leaves from *Msa* S1 plants—regenerants arising from transgenic callus event TG-20 (plant TG-20e) and isogenic wild-type were analyzed via confocal microscopy along with corresponding intact leaf images. **A** Donor plants wild-type (WT) and TG-20e. **B**–**E** On left, 3D images of leaf sections imaged by confocal microscopy; on right, corresponding leaf sections taken from plants. **B** Wild-type *Msa* S1. **C**–**E** Individual leaf phenotypes noted on plant TG-20e. All four confocal images were identical in width and height (441.94 μm × 441.94 μm), but the depth varied depending on the thickness of the leaf segment being imaged. *B* = 41.85 μm, *C* = 58.05 μm, *D* = 42.3 μm, *E* = 49.5 μm

Confocal microscopy was used to compare relative abundance of chlorophyll/chloroplasts (via autofluorescence) in cells of leaves that displayed all *lw1* mutant phenotypes generated in *Miscanthus* via CRISPR/Cas9. Leaves exhibited stripes (white or pale yellow), or were pale green/yellow or solid white. Striped leaves were of special interest to see if demarcations were distinct between green and white/pale-yellow stripes as had been noted in *lw1* mutants [41]. We observed leaves from two independent *Msa* S1 (4x) transformants (TG-20 and TG-25; both generated via biolistics) compared to isogenic wild-type *Msa* S1 (Fig. 3A). As expected, the confocal image of the wild-type leaf displayed clearly visible, densely packed, chloroplasts (green spherical structures) throughout the image (Fig. 3B). In comparison, chloroplasts in a TG-20 leaf appeared less densely packed and were lacking in certain sectors in the confocal image of a pale green/yellow *lw1* edited leaf (Fig. 3C). This lack of chloroplasts was further exaggerated in a solid white TG-20 leaf (Fig. 3E). A striped TG-20 leaf (Fig. 3D) displayed a similar lack of chloroplasts in two light-colored leaf areas (dark areas on left and right side of confocal image), whereas the green area (~ middle of image) had densely packed chloroplasts like that observed in wild-type. There also were clear demarcations between these areas that contained or lacked autofluorescence, as noted for *lw1* mutants [41].

To further explore the stripes in *lw1* edited leaves, *Msa* S1 transformation event TG-25c and TG-25h were imaged along with an isogenic wild-type. One leaf sample was taken from each corresponding plant and visualized via confocal (red chlorophyll/chloroplast autofluorescence) and bright field microscopy under different magnifications so direct comparisons could be made in areas where stripes were noted in those leaf sections (Additional file 1: Fig. S6). Lack of autofluorescence correlated with a white stripe (TG-25h) and mild autofluorescence with a pale-yellow stripe (TG-25c) compared to bright uniform autofluorescence in wild-type. Outside each stripe being tracked, the leaves were lighter green and displayed less, non-uniform autofluorescence compared to wild-type that displayed nearly uniform bright autofluorescence and dark green leaves (Additional file 1: Fig. S6). There was a clear demarcation between the white stripe and green areas flanking it (Additional file 1: Fig. S6E) that could also be observed in bright field images of that same leaf area.

### Sanger sequencing identified edits in multiple alleles and homeologs of *lemon white1*

**Fig. 4:**
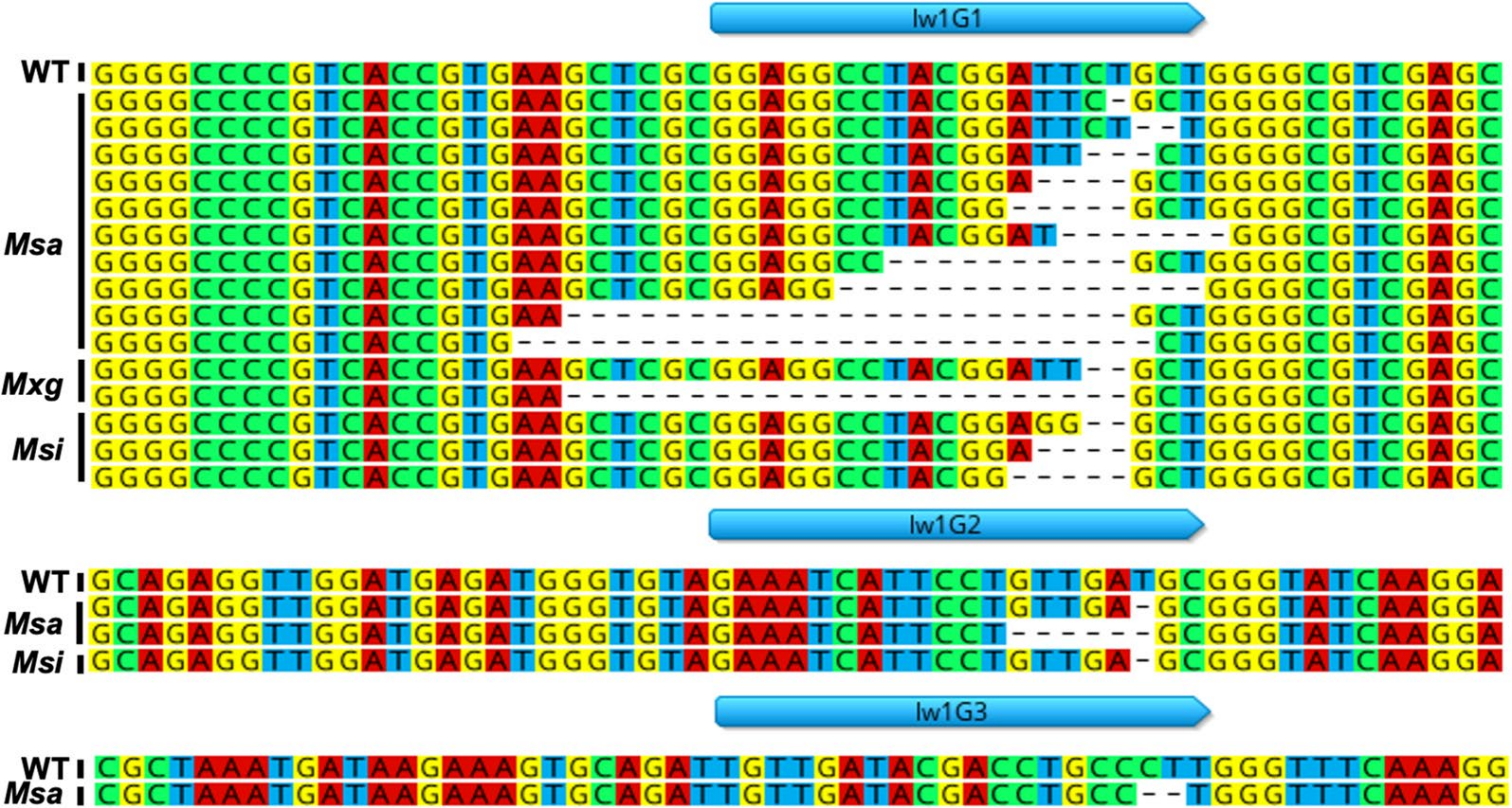
Sanger sequencing of *lw1* in transgenic edited and isogenic wild-type *Miscanthus* plants. A sequence alignment of the highly conserved region targeted by gRNAs lw1G1 (top panel), lw1G2 (middle panel) and lw1G3 (bottom panel), from edited and wild-type *Msa* S1, *M*x*g* Freedom, and *Msi* UI1. The gRNA is shown as a blue arrow above the alignment and deleted bases are marked with a hyphen (–). For the sake of simplicity, only one representative sequence of each type of deletion is shown. A complete alignment is available as aligned fasta files (Additional file 4)

One edited line per species was analyzed further and represented the ploidy range observed in *Miscanthus* [*Msi* UI1 (2x), *M*x*g* Freedom (3x), *Msa* S1 (4x)]. Successful editing was achieved in all three species at all *lw1* copies (homeologs and their alleles). Overall, 38.1%, 44.4% and 50.5% of the clones sequenced from edited *Msi* UI1, *M*x*g* Freedom and *Msa* S1, respectively, showed deletions (edits). Guide RNA lw1G1 yielded the largest number of deletions and were immediately adjacent to the predicted PAM site. This gRNA passed all filtering criteria (Fig. 1A) and was expected to successfully generate edits; predicted secondary structure of lw1G1 via UNAFold web server [42] (Additional file 1: Fig. S7A) indicated it would likely be highly effective [43, 44]. Deletions from lw1G1 ranged between 1 and 26 bp (Fig. 4). Separately, gRNAs lw1G2 and lw1G3 were responsible for deletions ranging from 1 to 6 bp, but the largest deletion (433 bp, in *Msa* S1) encompassed both target sites.

## Conclusions

We achieved our primary goal to demonstrate successful gene editing via CRISPR/Cas9 in *M*x*g* and its parental species, *Msa* and *Msi*, and targeted ploidies ranged from diploid to tetraploid. To the best of our knowledge, this is the first report of gene editing being achieved in *Miscanthus*, an important biomass crop. An initial screening of 87 *Miscanthus* genotypes identified substantial variation for embryogenic callus formation and regeneration, and a further subset showed variation for ability to be transformed via *A. tumefaciens* or biolistics—all factors that can affect gene editing efficiency. Optimized procedures were developed for five genotypes that included one *Msi* (2x), two *Msa* (2x and 4x) and one *M*x*g* (3x). A multi-step screening approach was devised to design gRNAs that could successfully target homeologs of a gene, advantageous for targeting genes in paleo-allopolyploid *Miscanthus*. The visual marker gene, *lw1*, targeted in maize to generate mutants via CRISPR/Cas9 [36–38], was selected for targeting in *Miscanthus*. Leaf phenotypes (pale green/yellow, striped, white) in edited *lw1* was a striking visual marker in *Miscanthus* to identify tissues/plants in the T0 generation for further analysis. This could be a good visual marker for other plant species when developing gene editing procedures. Sanger sequencing confirmed deletions in *lw1*. The generation of white leaves and white stripes in *lw1* edited *Miscanthus* plants confirmed that homeologs and their alleles were all successfully targeted. This proof of concept demonstrating that CRISPR/Cas9 could target and mutate homeologs of a specific gene in *Msa*, *Msi,* and *M*x*g* will enable improvement of this bioenergy crop by endogenous gene knockout or introduction of exogenous DNA at specific loci. Since deciphering gene function using conventional genetics is a major challenge in polyploid species, especially those that are obligate out-crossers like *Miscanthus*, this CRISPR/Cas9 tool could also be used to analyze the functions of specific genes via knockouts using our developed procedures.

## Materials and methods

### Screening *Miscanthus* germplasm for the ability to form embryogenic calli and regenerate shoots

Starting with immature inflorescences as explants, two research labs (in Alabama and Mississippi, U.S.) each focused on 1–2 species using in-lab procedures developed for their species of interest. Initial screenings were conducted on 87 genotypes that included *Msa* (43 genotypes), *Msi* (31 genotypes), and a group that included *M*x*g* and other species (13 genotypes) to determine which genotypes to focus on (Additional file 1: Table S1); immature inflorescences were harvested from the collection of Dr. Erik Sacks at the University of Illinois, and others from ornamental lines obtained from commercial nurseries. One genotype of *M*x*g* (MSU-MFL1; [45]) was harvested from Dr. Brian Baldwin’s research plot at Mississippi State University since it displays vigorous growth and high biomass yield (grown commercially as var. Freedom). All harvests took place in late spring–early summer before inflorescences emerged. Shoot tips were surface disinfected by two different methods: on a shaker (200 rpm), shaken in 70% ethanol for 5 min then in a 1.5% sodium hypochlorite (NaOCl) solution containing 0.1% Tween 20 for 20 min, or initially soaked in 75% ethanol for 20 min and then 6% NaOCl for 30 min. All shoot tips were rinsed in sterile demineralized (MilliQ) water. Outer leaf layers were removed and immature inflorescences were carefully dissected out and cut into 1.0 cm length explants.

In screening *Miscanthus* germplasm to select genotypes to genetically transform, immature inflorescences were placed on callus induction media (CIM; Table 1) and cultured in the dark at 25 °C. All media contained MS basal salts and vitamins [46]; media labeled as − 1 (used for *Msa* and *Msi*) contained additional myo-inositol (150 mg L^−1^) and media labeled as − 2 (used for *M*x*g*) contained additional thiamine-HCl (0.9 mg L^−1^). The common additive to all media (unless noted differently in Table 1) was 3% sucrose; − 1 media were adjusted to pH 5.8 solidified with phytagar (8.5 g L^−1^; Phytotechnology Labs) and − 2 media were adjusted to pH 5.5 and solidified with phytoblend (7 g L^−1^; Caisson Labs). Additional additives for − 1 media included casein hydrolysate (1 g L^−1^), L-proline (0.2 g L^−1^), copper sulfate (1.25 mg L^−1^), and MES (0.5 g L^−1^). Additional additives for -2 media included L-proline (2.88 g L^−1^; [47]) and magnesium chloride-hexahydrate (750 mg L^−1^; [13]). Tissues were primarily cultured on media in 100 × 20 mm petri plates. Shallower plates (100 × 15 mm) were used for short-term (< 7 days) manipulations of the cultures.

After culture initiation, immature inflorescence explants were transferred weekly to fresh CIM to reduce phenolic accumulation, and then transferred every 2–3 weeks until generation of embryogenic calli (took approx. 8 weeks on CIM). Embryogenic calli were then transferred to callus maintenance media (CMM; Table 1) and continued to be cultured in the dark for multiplication of embryogenic calli or transferred to regeneration media (RM; Table 1) for culture under a 16:8 h photoperiod (350 µmol m^−2^ s^−1^) at 25–26 °C until small shoots arose (took approx. 3–4 weeks). When shoots reached > 5 mm in height, they were teased away from the embryogenic callus clump and transferred to rooting media (RtM; Table 1) in sterile Solo cups with lids (Solo SD5 5 oz. clear plastic sundae cup #760SD5, DLR100-0090 sundae cup dome lid #760DLR100) or Magenta GA-7 vessels (Sigma-Aldrich). They were maintained in these vessels under a 16:8 h photoperiod until vigorous plantlets developed. Plantlets were transplanted into a commercial soilless mix, acclimatized for 2 weeks, then moved into a greenhouse for continued growth.

In genotype screenings, prior to transfer off CIM, calli generated from immature inflorescences taken from one plant per accession (one genotype assessed per accession) were evaluated for the percentage of calli pieces that were capable of generating embryogenic calli and rated based on response percentages (excellent = 81–100%, very good = 61–80%, good = 41–60%, fair = 10–40%, poor = 1–20%; Additional file 1: Table S1). To test regeneration responses for those genotypes that generated embryogenic calli, 8–12 embryogenic calli pieces (2–3 mm in diameter) were transferred to RM for shoot generation. After 4 weeks, calli were evaluated for the percentage that generated shoots and rated using the categories listed above.

Thirteen genotypes that gave acceptable regeneration responses (Additional file 1: Table S1) were screened for ability to be transformed using the *egfp* reporter gene (GFPGUSPlus, Addgene plasmid #64401). The five genotypes selected for use in gene editing experiments included *Msa* ‘Tohoku-2010-020’ (S1, 4x), *Msa* RU2012-110 (S13, 2x), *Msi* ‘Purpurascens’ (P1, 2x), *Msi* 10UI-008-2011-1-Row Replicated—CHA-115-7 (UI1, 2x), and *M*x*g* Freedom (3x). Newly generated embryogenic calli (from immature inflorescence explants) of these genotypes were used in transformations and were also maintained on CMM-1 (*Msa* and *Msi*) or CMM-2 (*M*x*g*; Table 1) for generation of additional embryogenic calli.

### Guide RNA design and vector construction

We used a combination of orthology relationships between sorghum and *Miscanthus* gene models [12] deposited in Phytozome [48] and OrthoFinder [49] to find the maize *lw1* orthologs in *Miscanthus* as well as sorghum. Since *Miscanthus* spp. are outbred, the alleles are rarely identical. Being paleo-allopolyploids, there are two homeologous genes that are likely to be functionally redundant. Therefore, targets in diploid *Miscanthus* included two homeologs and two alleles per homeolog to knock out *lw1* function. Triploids and tetraploids have three and four alleles per homeolog, respectively, to target (Additional file 1: Fig. S5). A reference genome is only available for one diploid *Msi* genotype, further complicating the design of gRNAs that targeted all *lw1* copies in genotypes besides the reference.

A multi-step pipeline was used to select gRNAs to generate deletions in *lw1* (Fig. 1A). We first identified regions that were highly conserved between both *Miscanthus* homeologs and sorghum; regions that are highly conserved across species and genera are more likely to be identical across different genotypes and, therefore, better targets for editing. Orthologs of *lw1* in the genic region of *Msi* ‘KS1’ and sorghum (Misin01G091700, Misin02G083200 and Sobic.001G102500, respectively) were aligned using MAFFT (Multiple Alignment using Fast Fourier Transform) v. 7.405 [50] with the following options: “–auto –op 1.53 –ep 0.123 –reorder” (Additional file 2). The “Find CRISPR Sites” tool from Geneious Prime 2019 [51] was used to search for all possible gRNA in the genic region of the *lw1* gene under default options with the exception that the specificity score was calculated against possible off-targets in *Msi* reference genome. The gRNAs were also identified using the Custom Alt-R^®^ CRISPR-Cas9 gRNA from IDT [52].

Predicted gRNAs (1452) were filtered based on our criteria that guides must target conserved exonic regions (443 of 1452) in the two *Msi* homeologous genes, as well as sorghum, to increase the probability of editing the *lw1* gene in multiple genotypes in the different *Miscanthus* species (Fig. 1A). This also increased the odds that a single gRNA will target the different copies of homeologous genes, thereby decreasing the number of gRNAs needed to edit all haplotypes of homeologous genes in diploid, triploid, and tetraploid *Miscanthus* species. The predicted gRNAs were then ranked based on their activity [53] and specificity scores [54] that reduced the number of potential gRNAs to 43 (of 443). After fusing the gRNA sequence with tracrRNA (gRNA::tracrRNA, also known as single guide RNA/sgRNA) the gRNA candidates that passed these filtering criteria were subjected to RNA secondary structure prediction to improve editing efficiency [55]. Secondary structure prediction of the sgRNAs was determined using mfold [42, 56], followed by filtering based on the criteria proposed by Liang et al. [43]. Briefly, filtering criteria focused on: (1) gRNA with GC% between 30 and 80%; (2) conserving the repeat and anti-repeat, 2nd, and 3rd stem loop structure of the sgRNA; (3) total base pairing < 13 and consecutive base pairing between gRNA and tracrRNA sequence < 8; (4) < 7 internal base pairing within the gRNA sequence.

Overall, three gRNAs representing the different filtering steps in the flowchart were chosen to collectively target exons 2 and 3 of the *lw1* genes in *Miscanthus*; lw1G1: 5′-GGAGGCCTACGGATTCTGCT-3′, lw1G2: 5′-GAAATCATTCCTGTTGATGC-3′, lw1G3: 5′-TGTTGATACGACCTGCCCTT-3′ (Additional file 1: Fig. S7; Additional file 2). Since the sequence for lw1G3 started with a thymine base, a single guanine base was added to its 5'-end to enable expression under a U6 promoter (Additional file 1: Fig. S7C) [57].

Each of the selected gRNAs was assembled between a monocot U6 promoter (upstream of the gRNA) and a tracrRNA sequence (downstream of the gRNA) thereby forming individual transcription units. A 1580 bp cassette containing the three selected gRNAs was synthesized by SynBio Technologies (Fig. 1B). The cassette was then cloned into the binary vector pPTN1399 using *Sma*I blunt digestion and ligation to create pHA194 (Additional file 1: Fig. S3; Additional file 3).

### Dose–response curve using paromomycin

The selectable marker gene in pHA194 was *nptII,* an aminoglycoside phosphotransferase that inactivates aminoglycoside antibiotics like kanamycin and paromomycin. Since most monocots have displayed varying degrees of resistance to kanamycin [58], paromomycin has been successfully used as a selection agent with *nptII* [59]. To identify the ideal concentration of paromomycin to incorporate into media to select *Miscanthus* calli that have been transformed with pHA194, a dose–response curve was generated by the addition of a range of paromomycin concentrations to CMM-1 culture medium. Per paromomycin concentration tested, 15 pieces of *Msi* UI1 calli 2–3 mm in diameter were individually weighed and plated on CMM-1 containing 0, 50, 75, 100, 125, 150, 175, and 200 mg L^−1^ paromomycin. After 3 weeks incubation in the dark, the embryogenic calli were individually weighed to determine average weight gain per callus piece and visually assessed for growth inhibition.

### Particle bombardment-mediated transformation

Particle bombardments were conducted using the Particle Delivery System PDS-1000/He (Bio-Rad). Embryogenic calli (1–2 mm pieces) were osmotically pre-treated on callus osmoticum medium (COM-1; Table 1) for 4 h, with embryogenic calli clustered in the center of the plate to form a 2.5-cm circle, bombarded at 1100 psi with a 8-cm target distance, then incubated on that medium in the dark overnight. DNA-gold microprojectiles were prepared following Bio-Rad’s instruction manual (Catalog #1652257) with a few modifications: a 25 μl aliquot of gold microprojectiles (1.0 μm size, 100 mg ml^−1^) suspended in 50% (*v*/*v*) sterile glycerol was combined with 25 μl DNA (3.6 μg pHA194 DNA in sterile MilliQ water), 50 μl 2.5 M CaCl_2_, and 20 μl 0.1 M spermidine in a 1.5 ml sterile microcentrifuge tube under constant vortexing at 1250 rpm for 10 min. The DNA-coated microprojectiles were pelleted by centrifugation at 5000 rpm for 1 min, washed with 150 μl 70% (*v*/*v*) ethanol, followed by 150 μl absolute ethanol wash, then resuspended in 60 μl absolute ethanol. The DNA-coated microprojectiles were placed back on a vortexer, and 10 μl were removed and placed on each macrocarrier for delivery via biolistics, with 0.6 μg DNA delivered per “shot”, one shot per plate of embryogenic calli.

Sixteen hours post-bombardment, embryogenic calli were transferred to callus selection medium (CSM-1; Table 1) and cultured in the dark for 2 weeks, then transferred to fresh CSM-1 and cultured an additional 2–3 weeks. Surviving embryogenic calli were transferred to regeneration selection medium (RSM-1; Table 1) and cultured under a 16:8 h photoperiod for 3 weeks. Regenerated shoots larger than 0.3 cm were transferred to rooting selection medium (RtSM-1; Table 1). After 2–3 weeks, plantlets taller than 0.5 cm with 2–3 roots were transplanted into a commercial soilless mix in a flat that was fitted with a clear dome cover to retain moisture, and acclimatized over the next 14 days then transferred to the greenhouse for continued growth.

### *Agrobacterium tumefaciens*-mediated transformation

Plasmid pHA194 (Additional file 1: Fig. S3) was electroporated into *A. tumefaciens* strain EHA105 [60] and used to transform *Miscanthus* embryogenic calli. An *A. tumefaciens* suspension was inoculated from frozen glycerol stock into 3 ml of liquid YEB medium containing 25 mg L^−1^ rifampicin, and incubated on an orbital shaker at 225 rpm in the dark overnight at 28 °C. Cell density was measured using a Nanodrop spectrophotometer (Thermo Scientific) at OD_600_. The needed volume for *OD*_600_ = 0.3 (*Msa* and *Msi*) or *OD*_600_ = 0.6 (*M*x*g*) was transferred to microcentrifuge tubes and centrifuged for 2 min at 9000 rpm. Bacterial pellets were resuspended in co-culture media CCM-1 (*Msa* and *Msi*) or CCM-2 (*M*x*g*; Table 1). *A. tumefaciens* used in *M*x*g* transformations were pre-incubated in CCM-2 on a shaker at 22–24 °C for 30 min at 110 rpm.

Embryogenic calli were added to *A. tumefaciens* cultures in vessel sizes that were dependent on the number of embryogenic calli to be incubated/co-cultured; they were incubated for 20 min (*Msa* & *Msi*) or 30 min (*M*x*g*) in the dark at 22–24 °C on a shaker at 110–120 rpm. Embryogenic calli were removed and blotted on sterile Kimwipes, then transferred to petri plates containing sterile Whatman filter paper moistened with liquid CCM and co-cultured for 7 days (*Msa* & *Msi*) or 5 days (*M*x*g*) in the dark at 22–24 °C. Embryogenic calli were washed 2–3 times with sterile MilliQ water, then with a final rinse containing 100 mg L^−1^ timentin (*Msa* & *Msi*) or 1 g L^−1^ cefotaxime (*M*x*g*). Embryogenic calli were blotted with sterile Kimwipes, then transferred to recovery media (RCM-1 or RCM-2; Table 1) and cultured in the dark for 7 days.

For *Msa* and *Msi*, selection and regeneration followed the procedure described for biolistics, but 100 mg L^−1^ timentin was included in the media (CSM-1 T, RSM-1 T; Table 1) until *A. tumefaciens* could no longer be detected. For *M*x*g*, embryogenic calli were placed on callus selection medium (CSM-2; Table 1) for two cycles (each cycle was 2 weeks) and cultured in the dark. They were transferred to regeneration selection medium (RSM-2; Table 1) and grown for four cycles under a 16:8 h photoperiod, with cycles 2–4 containing 50 mg L^−1^ paromomycin instead of 100 mg L^−1^. If additional shoot regeneration cycles were needed, they were transferred to RM-2 (no selection). Regenerated shoots were transferred to RtM-2 medium for rooting. Plantlets with vigorous root systems were transplanted into a commercial soilless mix and acclimatized as described above.

### Detecting *nptII* in putative transgenic plantlets

Polymerase chain reaction (PCR) to detect the *nptII* transgene was conducted on DNA extracted from 1 mm pieces of putative transgenic plantlets using the Platinum^™^ Direct PCR Universal Master Mix (Catalog # A44647500). Primers NPT-F (5′-GATTGAACAAGATGGATTGCACGCAGGTT-3′) and NPT-R (5′-CTCTTCAGCAATATCACGGGTAGCCAA-3′) were used to amplify a 691 bp segment of *nptII*. Isogenic wild-type plants were used as negative controls and pHA194 was used as a positive control. Each 1 mm piece of plant tissue was heated at 98 °C for 1 min in the lysis buffer according to manufacturer's instructions, and 1 μl of the tissue lysate was transferred to the PCR master mix provided in the kit. An initial denaturation step at 94 °C for 2 min was followed by 35 cycles of denaturing, annealing and amplification (94 °C for 15 s, 60 °C for 15 s, 68 °C for 20 s). Samples of amplified DNA were run on a 1% agarose gel in 1X TAE buffer for visualization. Detection of *nptII* in larger plants was carried out using the NPTII ELISA kit (Agdia, PSP 73000), following manufacturer’s instructions.

### Visual detection of *lw1* loss of function in leaf tissues and plants

Three leaves from one *Msa* S1 *lw1* edited plant (TG-20e) that showed different phenotypes (pale green/yellow, striped, white) and one from an isogenic wild-type plant were viewed via confocal microscopy. Edited TG-20e arose from transformation event TG-20 (callus piece 20) that had been transformed/edited via biolistics. Images were acquired on Nikon Instruments point scanning Ti2-A1R confocal microscope using 40 × 1.25NA water immersion objective. Two GaAsP detectors were used to capture the green and red signals. Corresponding leaf section and plant images were captured using a smartphone camera.

### Sanger sequencing to confirm edits in the *lw1* gene

PCR primers (A0159F 5′-CAGGCGATGTGATCAAGACG-3′ and A0160R 5′-GTTGAGCTAGACCCATCAAGT-3′) were designed using Geneious Prime to amplify a highly conserved 1128 bp region of both homeologs of the *lw1* gene. This region is targeted by gRNA lw1G1, lw1G2 and lw1G3. DNA was extracted from leaves that showed the wild-type phenotype (green) as well as those that exhibited white stripes, were pale green/yellow, or completely white. PCR was performed using the Platinum Direct PCR Universal Master Mix (Invitrogen, A44647100). Amplified fragments were gel extracted and purified using QIAquick Gel Extraction Kit (Qiagen, 28707). The Zero Blunt TOPO PCR Cloning Kit (Invitrogen, 2422133) and TOP10 (Invitrogen, C66411) electrocompetent *E. coli* cells were used for cloning. Amplicons were cloned to separate out the different copies and to account for the chimeric nature of the edits in T0 lines.

A total of 25 *E. coli* colonies containing the *lw1* amplicons were picked from the *Msi* UI1 isogenic wild-type and 21 clones from an edited *Msi* UI1 line. Similarly, 28 colonies from the isogenic wild-type and 170 colonies from the edited *Msa* S1, and 36 colonies from the isogenic wild-type and 27 colonies from the *lw1* edited *M*x*g* Freedom were chosen for plasmid preps. Plasmids were extracted from overnight cultures of single colonies and purified using QIAprep Spin Miniprep Kit (Qiagen, 27106). Plasmids were Sanger sequenced at MCLAB (San Francisco, CA) using four primers (two in vector and two in conserved regions of *lw1*) to sequence with enough overlap; primers included M13 Forward (− 20) (5′-GTAAAACGACGGCCAG-3′), M13 Reverse (5′-CAGGAAACAGCTATGAC-3′), A0159F, and A0160R. Sequences were end trimmed to remove low quality bases (5% error probability), and vector sequences were removed using UniVec (High Sensitivity) in Geneious Prime. For each clone, a consensus sequence was generated. For each genotype (*Msa*, *Msi*, *M*x*g*) an alignment of the consensus of the wild-type sequence and that of the edited lines was obtained (Additional file 4). A subset of these, showing one instance of every type of edit, is shown in Fig. 4.

## Supplementary Information

**Additional file 1: Table S1.** *Miscanthus* lines screened in vitro for specific characteristics. **Figure S1.** Examples of *Miscanthus* calli, regenerants and transformants generated in initial genotype screenings. **Figure S2.** Dose–response curve for *Miscanthus* using paromomycin. **Figure S3.** Map of plasmid pHA194. **Figure S4.** PCR screening of putative *lw1* edited *Miscanthus* plants, amplifying *nptII *in all five genotypes.** Figure S5.** A graphical representation of gene/allele copy number, specifically *lemon white1 *(*lw1*), in paleo-allopolyploid *Miscanthus*.** Figure S6.** Confocal and bright field images of *M. sacchariflorus* S1 TG-25 leaves: wild-type vs. *lw1* edited. **Figure S7.** Secondary structure prediction of three *lw1* single guide RNA (sgRNA) designs. Description: Supplemental table and figures; format: PDF.

**Additional file 2.** Sequence and location of three gRNAs in the *lemon white1* gene. Description: Multiple sequence alignments of *lemon white1* orthologs from sorghum and miscanthus and the three gRNAs used to edit the gene; format: FASTA multiple sequence alignment.

**Additional file 3.** Sequence and detailed map of pHA194. Description: Sequence and annotation of all the parts in pHA194 plasmid; format: GenBank sequence format.

**Additional file 4.** Sequence data for edits in *lemon white1* in three *Miscanthus* species. Description: Aligned DNA sequence; format: FASTA.

## Data Availability

All data generated or analyzed during this study are included in this published article (and its supplementary information files).
