# Supplementary material for: Transformation and gene editing in the bioenergy grass Miscanthus
Source: Biotechnol Biofuels Bioprod. 2022 Dec 28;15:148. doi: 10.1186/s13068-022-02241-8 (PMC9798709; doi:10.1186/s13068-022-02241-8)
Supplement: Supplementary file 1 — Additional file 1: Table S1. Miscanthus lines screened in vitro for specific characteristics. Figure S1. Examples of Miscanthus calli, regenerants and transformants generated in initial genotype screenings. Figure S2. Dose–response curve for Miscanthus using paromomycin. Figure S3. Map of plasmid pHA194. Figure S4. PCR screening of putative lw1 edited Miscanthus plants, amplifying nptII in all five genotypes. Figure S5. A graphical representation of gene/allele copy number, specifically lemon white1 (lw1), in paleo-allopolyploid Miscanthus. Figure S6. Confocal and bright field images of M. sacchariflorus S1 TG-25 leaves: wild-type vs. lw1 edited. Figure S7. Secondary structure prediction of three lw1 single guide RNA (sgRNA) designs. Description: Supplemental table and figures; format: PDF. [file 13068_2022_2241_MOESM1_ESM.pdf]

Table S1. *Miscanthus* lines screened in vitro for specific characteristics.

Entry	Accession #	Source(s)	Callus	Callus quality*	Regeneration **	Transformation efficiency (%)***	Lines used to test editing	Abreviations used in the manuscript
Miscanthus sinensis '10UI-008-2011-1-Row Replicated - CHA-115-7'	10UI-008-2011-1-Row Replicated - CHA-115-7	University of Illinois	Yes	Very good	Very good	3.7 (Biolistics)	**Yes**	**UI1**
Miscanthus sinensis 'Purpurascens, Flame Grass'	Msi-P2	Sow_Blessed_Seeds	Yes	Very good	Very good	4 (Agrobacterium)	No	P2
Miscanthus sinensis 'PMS-014'	PMS-014	Wuhan Botanical Garden	Yes	Very good	Very good	3 (Agrobacterium)	No	PMS-014
Miscanthus sinensis 'Outdsidepride'	Msi-O1	Outsidepride Seeds, LLC	Yes	Good	Very good	0 (Agrobacterium)	No	O1
Miscanthus sinensis 'Outdsidepride New Hybrid'	Msi-ONH1	Outsidepride Seeds, LLC	Yes	Good	Very good	0 (Agrobacterium)	No	ONH1
Miscanthus sinensis 'Silberfeder (Silver Feather)'	UI10-00085	Bluemel	Yes	Good	Good	Not tested	No	
Miscanthus sinensis 'Undine'	Msi-UN1	HudsonAlpha	Yes	Good	Fair	0 (Agro and biolistics)	No	UN
Miscanthus sinensis 'US47-0011'	CANE9233	USDA-NPGS	Yes	Good	Not tested	Not tested	No	US47
Miscanthus sinensis 'PMS-009'	PMS-009	WBG	Yes	Fair	Good	0 (Biolistics)	No	PMS-009
Miscanthus sinensis 'Malepartus'	UI12-00002	Walla Walla	Yes	Fair	Fair	Not tested	No	
Miscanthus sinensis 'Onna-1b'	Onna-1b	University of Illinois	Yes	Fair	Fair	Not tested	No	
Miscanthus sinensis 'Onna-1a'	Onna-1a	University of Illinois	Yes	Fair	Fair	Not tested	No	
Miscanthus sinensis 'Grosse Fontaine'	UI10-00053	Bluemel	Yes	Fair	Fair	0 (Agro and biolistics)	No	
Miscanthus sinensis 'KS1'	Msi-KS1	Polish Academy of Sciences	Yes	Fair	Fair	0 (Agro and biolistics)	No	KS1
Miscanthus sinensis 'NC-2010-001 Nat Pop 2011-3-2-8'	UI14-00001	University of Illinois	Yes	Fair	Poor	Not tested	No	
Miscanthus sinensis 'Purpurascens, Flame Grass'	Msi-P1	Sow_Blessed_Seeds	Yes	Good	Good	5.7 (Agrobacterium)	**Yes**	**P1**
Miscanthus sinensis 'Silberturm (Silver Tower)'	UI10-00088	Bluemel	Yes	Poor	Poor	Not tested	No	
Miscanthus sinensis 'Gracillimus'	PI295764	USDA-NPGS	Yes	Poor	Poor	Not tested	No	
Miscanthus sinensis var. condensatus 'Cosmopolitan'	UI10-00015	ECG	Yes	Poor	Poor	Not tested	No	
Miscanthus sinensis 'Kaskade' (Digging Dog)	UI11-00014	Juvik	No	N/A	N/A	N/A	N/A	
Miscanthus sinensis 'Grosse Fontaine 4x'	UI11-00008	Juvik	No	N/A	N/A	N/A	N/A	GF
Miscanthus sinensis 'November Sunset'	UI10-00075	Bluemel	No	N/A	N/A	N/A	N/A	
Miscanthus sinensis 'Variegatus'	UI10-00097	ECG	No	N/A	N/A	N/A	N/A	
Miscanthus sinensis 'Blondo'	UI10-00030	Walla Walla	No	N/A	N/A	N/A	N/A	
Miscanthus sinensis ssp. condensatus 'US64-0007-01'	PI294605	USDA-NPGS	No	N/A	N/A	N/A	N/A	
Miscanthus sinensis 'US64-0004-02'	PI294602	USDA-NPGS	No	N/A	N/A	N/A	N/A	
Miscanthus sinensis 'Strictus'	UI10-00092	Bluemel	No	N/A	N/A	N/A	N/A	
Miscanthus sinensis 'PMS-038'	PMS-038	WBG	No	N/A	N/A	N/A	N/A	
Miscanthus sinensis 'Hokkaido Univ-selection-4'	JPN-2011-024	HU	No	N/A	N/A	N/A	N/A	
Miscanthus sinensis 'Miyazaki'	UI11-00001.5	Japan	No	N/A	N/A	N/A	N/A	
Miscanthus sinensis 'PF20014.001'	UI11-00029	NEF	No	N/A	N/A	N/A	N/A	
Miscanthus sinensis 'Emerald Shadow' x FP1-2	13UI-013R-36	University of Illinois	Yes	Fair	Fair	Not tested	No	
Miscanthus x giganteus 3x '12UI-001-007'	12UI-001-007	UI	Yes	Fair	Fair	Not tested	No	
Miscanthus x giganteus 3x	10UI-032-001	University of Illinois	Yes	Fair	Fair	Not tested	No	
Miscanthus x giganteus 3x 'MSU-MFL1', 'Freedom'	MSU-MFL1	Mississippi State University	Yes	Fair	Fair	1-2 (Agrobacterium)	**Yes**	
Miscanthus sinensis x Miscanthus sacchariflorus BC 'Andante'	UI10-00023	Bluemel	Yes	Fair	Fair	Not tested	No	
Miscanthus floridulus 'US56-0022-03'	PI230189	USDA-NPGS	Yes	Poor	Poor	Not tested	No	
Miscanthus floridulus 'PI295762'	PI295762	USDA-NPGS	Yes	Poor	Poor	Not tested	No	
Miscanthus x giganteus 3x '64-1'	UI10-00123		No	N/A	N/A	N/A	N/A	
Miscanthus sinensis x Miscanthus sacchariflorus 2x 'Mt. Washington'	UI10-00072	Long's Garden	No	N/A	N/A	N/A	N/A	
Miscanthus 'M75-062'	PI423566	USDA-NPGS	No	N/A	N/A	N/A	N/A	
Miscanthus 'US64-0016-03'	PI302423	USDA-NPGS	No	N/A	N/A	N/A	N/A	
Miscanthus sinensis Kaskade x M. sacchariflorus 4x 'Tohuku'	13UI-008-R-001	University of Illinois	No	N/A	N/A	N/A	N/A	
Miscanthus x giganteus 4x	13UI-013R-66	University of Illinois	No	N/A	N/A	N/A	N/A	
Miscanthus sacchariflorus 4x 'Tohoku-2010-020'	JPN-2010-008	HU	Yes	Very good	Very good	9 (Biolistics)	**Yes**	**S1**
Miscanthus sacchariflorus 'RU2012-081'	RU2012-081	USDA-VIR****	Yes	Very good	Very good	Not tested	No	S4
Miscanthus sacchariflorus 'RU2012-110'	RU2012-110	USDA-VIR	Yes	Good	Good	5.7 (Agrobacterium)	**Yes**	**S13**
Miscanthus sacchariflorus 4x 'EMI-5'	UI11-00027	USDA-Canal Point	Yes	Good	Good	Not tested	No	S2
Miscanthus sacchariflorus 'Robustus-Bluemel'	UI10-00009	Bluemel	Yes	Good	Fair	Not tested	No	RB
Miscanthus sacchariflorus 'RU2012-062'	RU2012-062	USDA-VIR	Yes	Fair	Fair	Not tested	No	
Miscanthus sacchariflorus 'RU2012-179'	RU2012-179	USDA-VIR	Yes	Fair	Fair	Not tested	No	S3
Miscanthus sacchariflorus 'RU2012-182'	RU2012-182	USDA-VIR	Yes	Fair	Fair	Not tested	No	
Miscanthus sacchariflorus 'RU2012-185'	RU2012-185	USDA-VIR	Yes	Fair	Fair	Not tested	No	S7
Miscanthus sacchariflorus ssp. lutarioriparius 'PF30022'	UI11-00031	NEF	Yes	Poor	Poor	Not tested	No	
Miscanthus sacchariflorus 4x 'Bluemel'	UI10-00117	Bluemel	No	N/A	N/A	N/A	N/A	
Miscanthus sacchariflorus 4x 'Gotemba Gold'	UI11-00005	Glasshouse Works	No	N/A	N/A	N/A	N/A	
Miscanthus sacchariflorus 4x 'JM11-019'	JM11-019	HU	No	N/A	N/A	N/A	N/A	
Miscanthus sacchariflorus 4x 'Hokkaido Univ-selection-3'	JPN-2011-023	HU	No	N/A	N/A	N/A	N/A	
Miscanthus sacchariflorus 'RU2012-016'	RU2012-016	USDA-VIR	No	N/A	N/A	N/A	N/A	
Miscanthus sacchariflorus 4x 'RU2012-023'	RU2012-023	USDA-VIR	No	N/A	N/A	N/A	N/A	
Miscanthus sacchariflorus 'RU2012-025'	RU2012-025	USDA-VIR	No	N/A	N/A	N/A	N/A	
Miscanthus sacchariflorus 'RU2012-027'	RU2012-027	USDA-VIR	No	N/A	N/A	N/A	N/A	
Miscanthus sacchariflorus 'RU2012-036'	RU2012-036	USDA-VIR	No	N/A	N/A	N/A	N/A	
Miscanthus sacchariflorus 'RU2012-038'	RU2012-038	USDA-VIR	No	N/A	N/A	N/A	N/A	
Miscanthus sacchariflorus 'RU2012-044'	RU2012-044	USDA-VIR	No	N/A	N/A	N/A	N/A	
Miscanthus sacchariflorus 'RU2012-049'	RU2012-049	USDA-VIR	No	N/A	N/A	N/A	N/A	
Miscanthus sacchariflorus 4x 'RU2012-055'	RU2012-055	USDA-VIR	No	N/A	N/A	N/A	N/A	

Miscanthus sacchariflorus 'RU2012-059'	RU2012-059	USDA-VIR	No	N/A	N/A	N/A	N/A	
Miscanthus sacchariflorus 'RU2012-064'	RU2012-064	USDA-VIR	No	N/A	N/A	N/A	N/A	
Miscanthus sacchariflorus 'RU2012-068'	RU2012-068	USDA-VIR	No	N/A	N/A	N/A	N/A	
Miscanthus sacchariflorus 'RU2012-070'	RU2012-070	USDA-VIR	No	N/A	N/A	N/A	N/A	
Miscanthus sacchariflorus 'RU2012-082'	RU2012-082	USDA-VIR	No	N/A	N/A	N/A	N/A	
Miscanthus sacchariflorus 'RU2012-087'	RU2012-087	USDA-VIR	No	N/A	N/A	N/A	N/A	
Miscanthus sacchariflorus 'RU2012-096'	RU2012-096	USDA-VIR	No	N/A	N/A	N/A	N/A	
Miscanthus sacchariflorus 'RU2012-097'	RU2012-097	USDA-VIR	No	N/A	N/A	N/A	N/A	
Miscanthus sacchariflorus 'RU2012-101'	RU2012-101	USDA-VIR	No	N/A	N/A	N/A	N/A	
Miscanthus sacchariflorus 'RU2012-126'	RU2012-126	USDA-VIR	No	N/A	N/A	N/A	N/A	
Miscanthus sacchariflorus 'RU2012-136'	RU2012-136	USDA-VIR	No	N/A	N/A	N/A	N/A	
Miscanthus sacchariflorus 'RU2012-144'	RU2012-144	USDA-VIR	No	N/A	N/A	N/A	N/A	
Miscanthus sacchariflorus 'RU2012-145'	RU2012-145	USDA-VIR	No	N/A	N/A	N/A	N/A	
Miscanthus sacchariflorus 'RU2012-151'	RU2012-151	USDA-VIR	No	N/A	N/A	N/A	N/A	
Miscanthus sacchariflorus 'RU2012-153'	RU2012-153	USDA-VIR	No	N/A	N/A	N/A	N/A	
Miscanthus sacchariflorus 'RU2012-165'	RU2012-165	USDA-VIR	No	N/A	N/A	N/A	N/A	
Miscanthus sacchariflorus 'RU2012-197'	RU2012-197	USDA-VIR	No	N/A	N/A	N/A	N/A	
Miscanthus sacchariflorus 'RU2012-203'	RU2012-203	USDA-VIR	No	N/A	N/A	N/A	N/A	
Miscanthus sacchariflorus 'RU2012-206'	RU2012-206	USDA-VIR	No	N/A	N/A	N/A	N/A	
Miscanthus sacchariflorus 'RU2012-209'	RU2012-209	USDA-VIR	No	N/A	N/A	N/A	N/A	

Contact for inquiries about this germplasm collection - esacks@illinois.edu

Ratings and corresponding colors relate to percentage of explants able to generate embryogennic calli, or percentage of embryogenic calli capable of shoot regeneration

Poor (orange): 1-20% responded
Fair (yellow): 21-40% responded
Good (blue): 41-60% responded
Very good (green): 61-80% responded
Excellent (none rated in this category): 81-100% responded

*Callus quality referred to percentage of calli that were embryogenic

**Regeneration referred to percentage of calli capable of shoot regeneration; 8-12 embryogenic calli (2-3 mm diameter) were transferred to regeneration media (RM; Table 1) for shoot regeneration, and assessed for ability to generate shoots after 4 weeks

***Efficiency based on reporter gene expression

****USDA-VIR Collection Expedition 2012

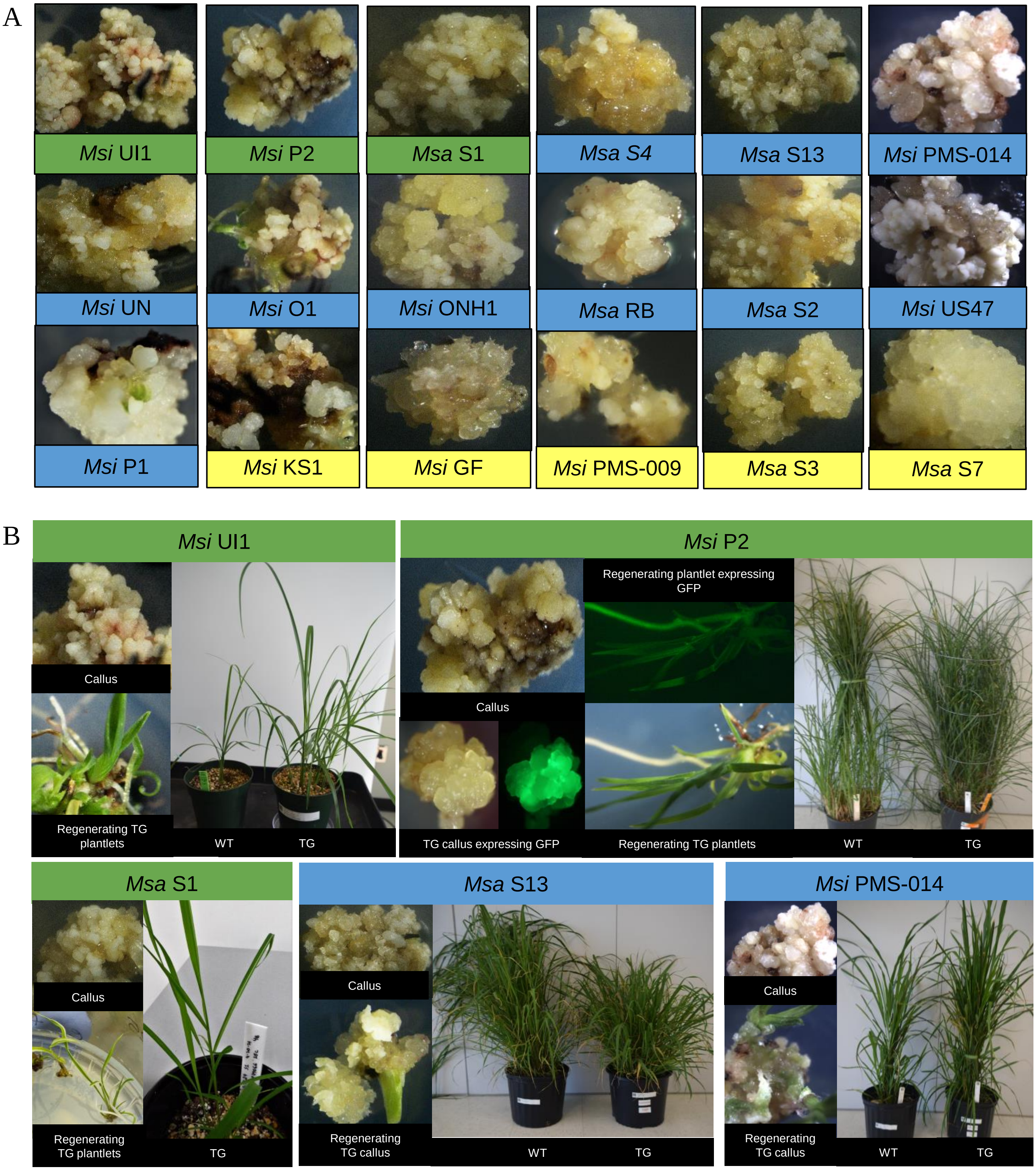

Fig. S1. Examples of *Miscanthus* calli, regenerants and transformants generated in initial genotype screenings. (A) Calli generated from immature inflorescences; 18 genotypes shown. (B) Genotypes were assessed for ability to regenerate shoots, and some were also assessed for ability to be transformed via biolistics or *A. tumefaciens*, screening for GFP expression. Footer/header colors referred to classifications based on percentage of calli that were embryogenic or percentage of calli capable of shoot regeneration: green=very good response (61-80%), blue=good response (41-60%), yellow=fair response (21-40%). TG=transgenic tissues/plants, WT=wild-type.

A

B

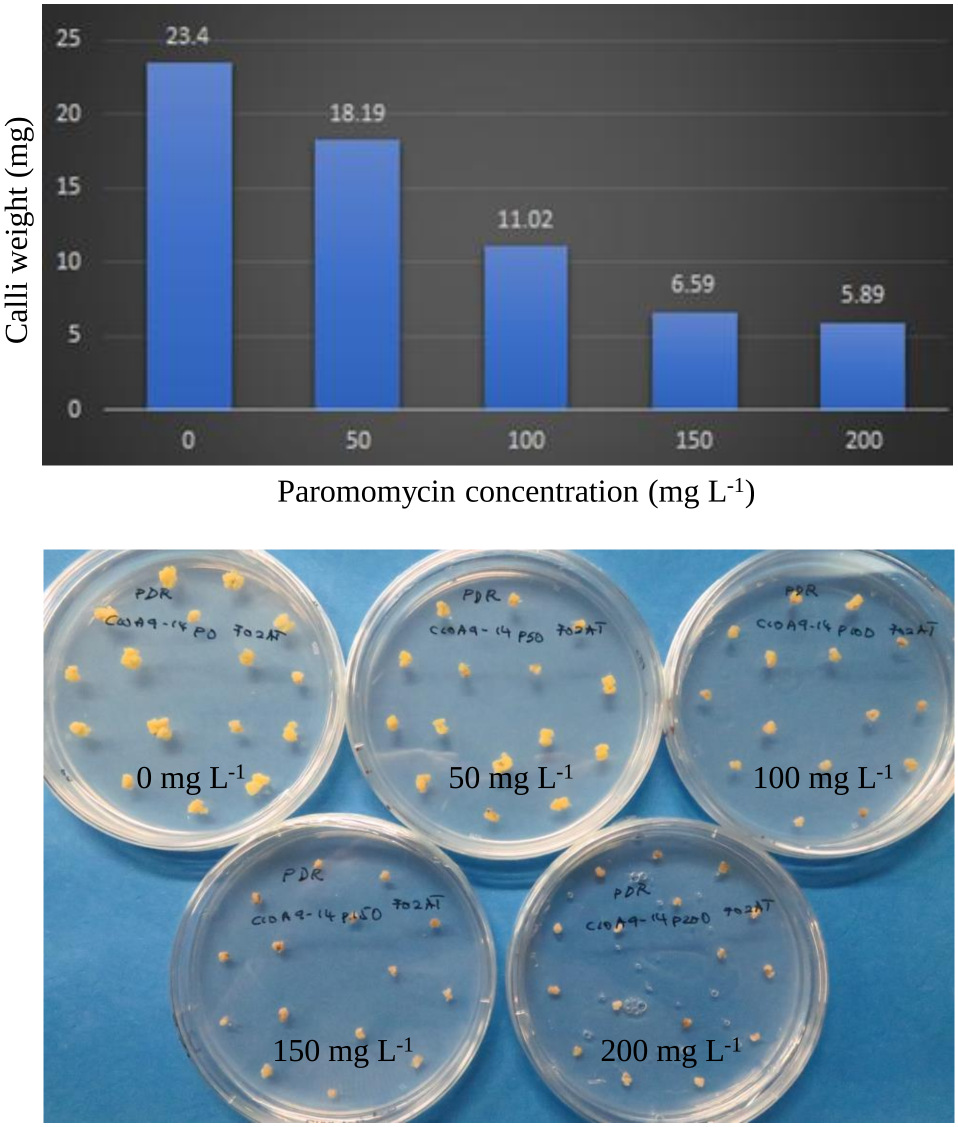

Fig. S2. Dose-response curve for *Miscanthus* using paromomycin.
Per treatment, 15 pieces of *Msi* UI1 calli ~2-3 mm in diameter were individually weighed then plated on CMM-1 + paromomycin at the following concentrations: 0, 50, 75, 100, 125, 150, 175, and 200 mg L^{-1}. After 3 wk incubation in the dark, the EC were individually weighed and visually evaluated for growth inhibition. (A) Average weight gain per callus on media containing different paromomycin concentrations. (B) Visual appearance of calli after 3 wk incubation on media containing paromomycin.

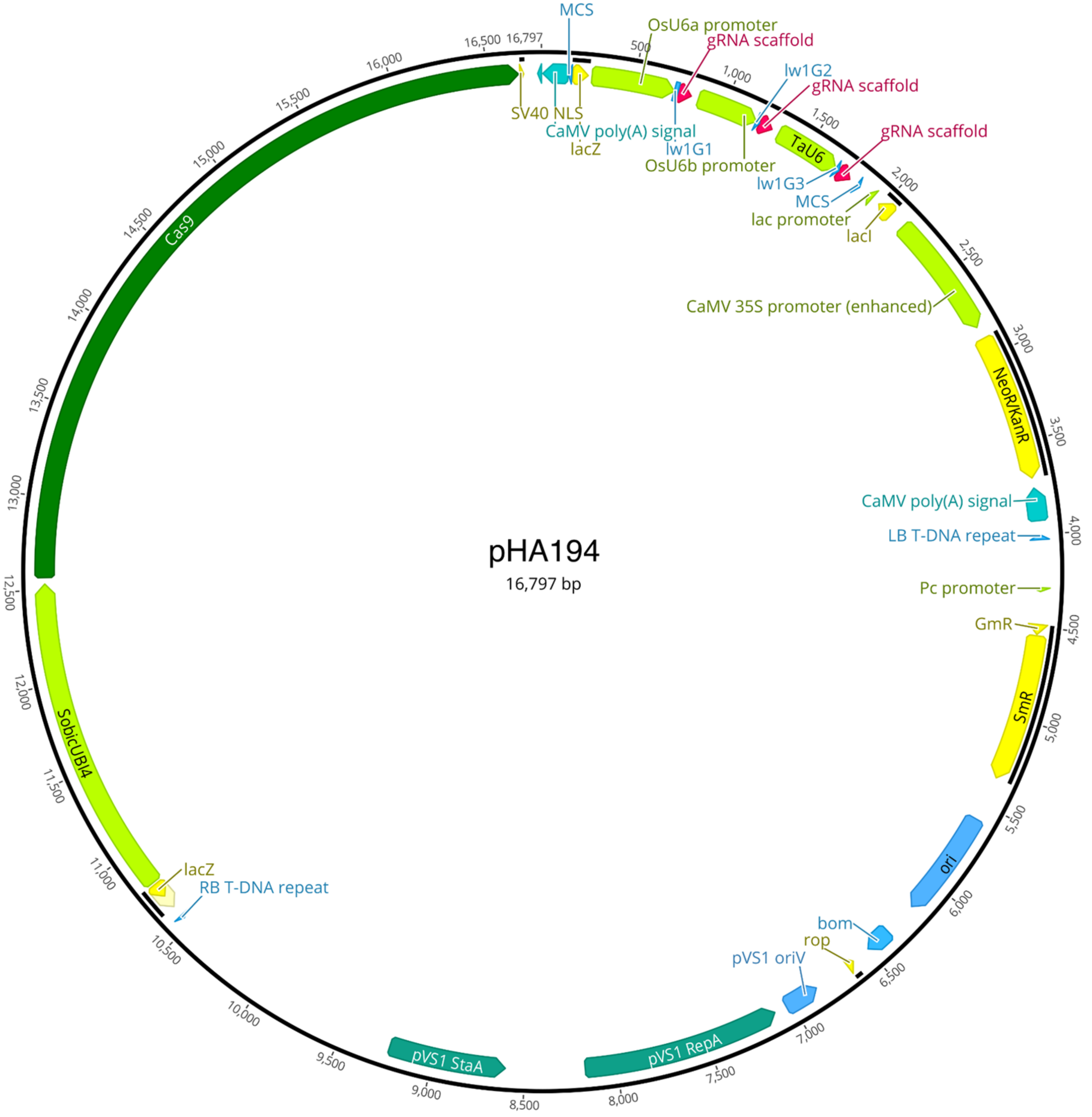

Fig. S3. Map of plasmid pHA194.

SobicUBI4, sorghum ubiquitin promoter; Cas9, sorghum codon-optimized Cas9; SV40 NLS, nuclear localization signal; CaMV poly(A) signal, terminator and poly(A) signal; OsU6a, OsU6b, & TaU6, class/type III RNA polymerase III promoters; gRNA scaffold, tracrRNA backbone sequence; NeoR/KanR, kanamycin/paromomycin resistance marker; SmR, spectinomycin resistance marker. Refer to Additional file 4.

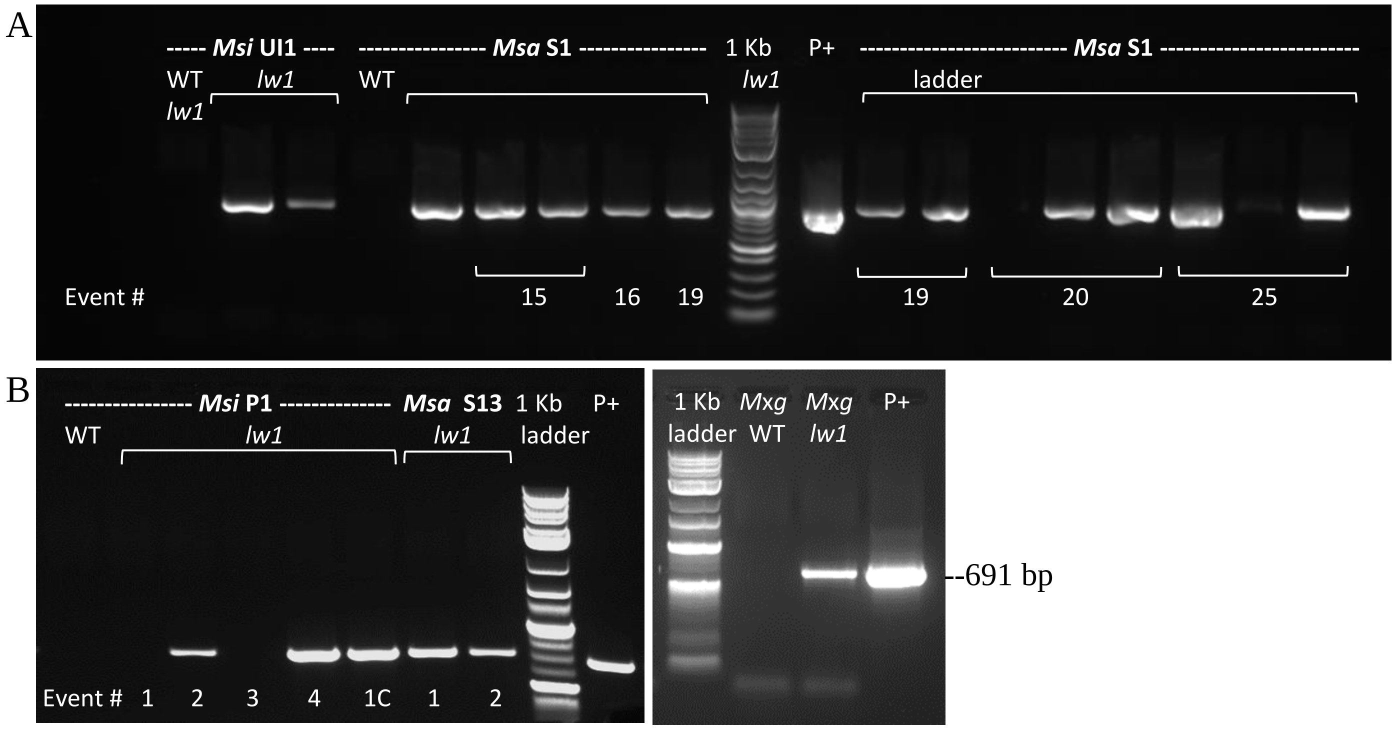

Fig. S4. PCR screening of putative *lw1* edited *Miscanthus* plants, amplifying *nptII* in all five genotypes. Leaf samples were harvested from putative transgenic plants and isogenic wild-type plants. Extracted DNA were amplified via PCR using *nptII* primers, then analyzed via agarose gel electrophoresis. (A) Gel showing *nptII* amplification in *Msi* UI1 and *Msa* S1 plant samples. (B) Gels showing *nptII* amplification in *Msi* PI, *Msa* S13 and *Mxg* Freedom plant samples. Abbreviations: WT=wild-type, *lw1*=putative gene-edited plants, P+=positive control (pHA194). Event # indicates transgenic plant(s) arose from that individual (numbered) callus piece. Plants arising from different calli were independent transformants.

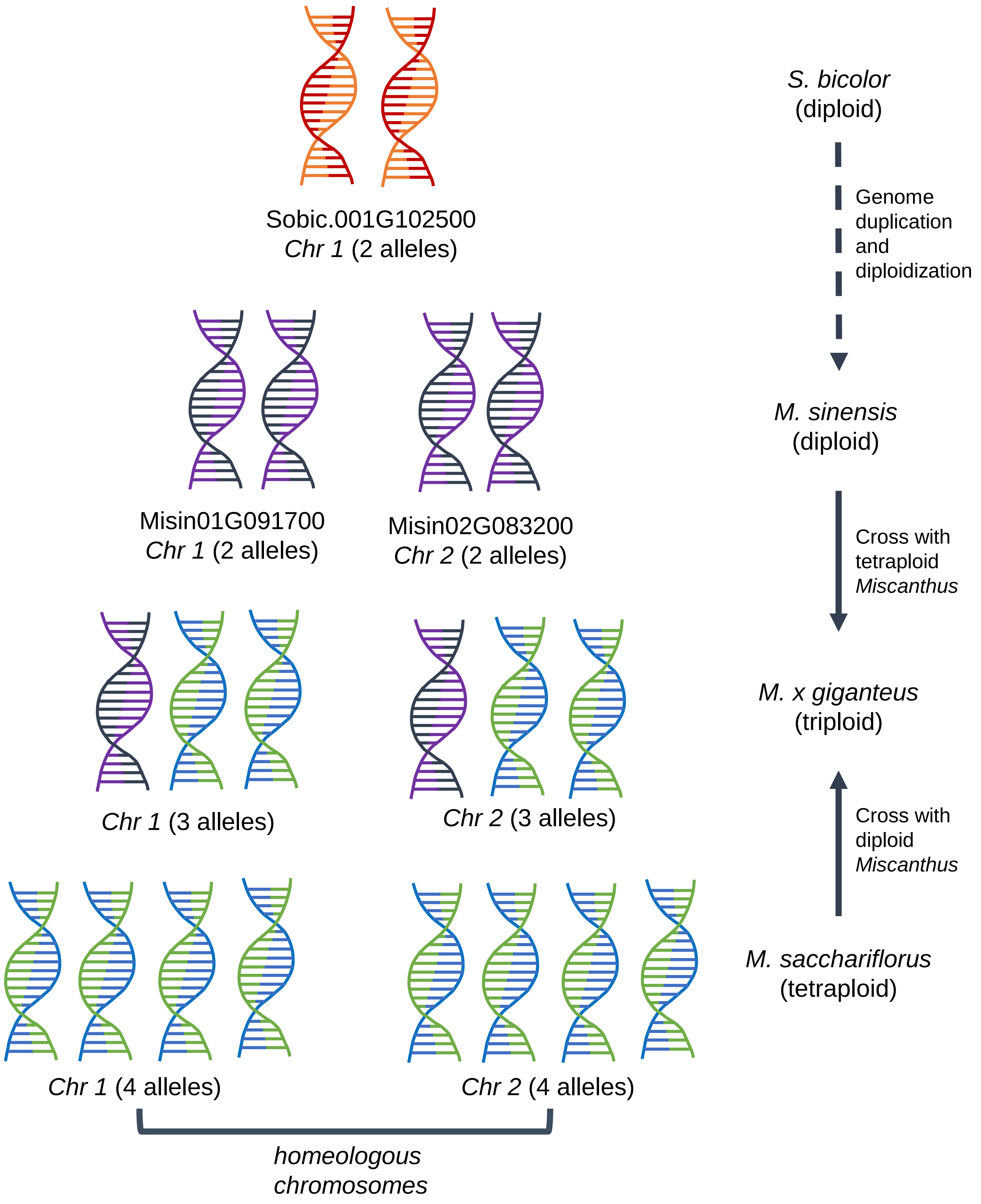

Fig. S5. A graphical representation of gene/allele copy number, specifically *lemon white1* (*lw1*), in paleo-allopolyploid *Miscanthus*.
The number of *lw1* alleles that need to be targeted to get complete loss of function mutants in *Miscanthus* ranges from four to eight. The *lw1* ortholog in sorghum resides on chromosome (Chr) 1. In *Miscanthus* the *lw1* orthologs reside on homeologous Chr 1 and Chr 2. In diploid *Miscanthus* genotypes (all *M. sinensis* and some *M. sacchariflorus*), there are two alleles per chromosome, resulting in four nearly identical copies of *lw1*. Similarly, tetraploid *M. sacchariflorus* genotypes have eight copies of *lw1*. The triploid hybrid *M.* x *giganteus*, a cross between a tetraploid *M. sacchariflorus* and diploid *M. sinensis*, has six copies of *lw1*.

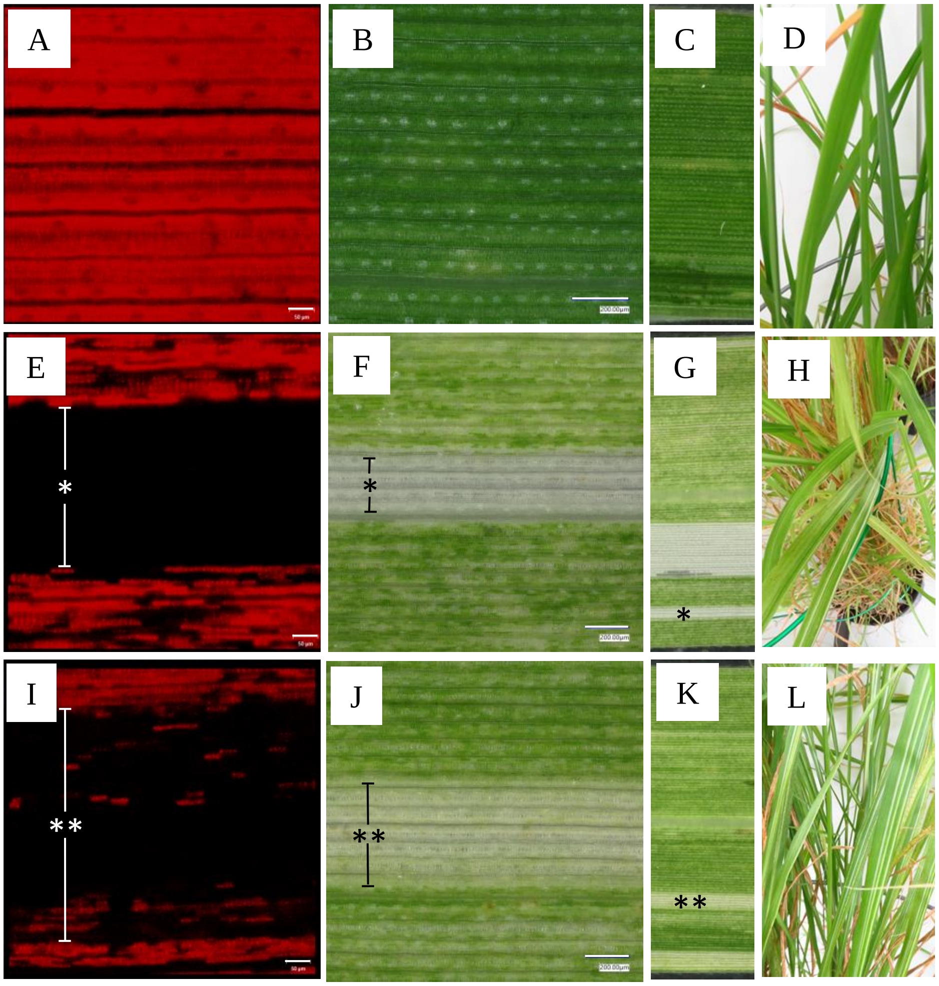

Fig. S6. Confocal and bright field images of *M. sacchariflorus* S1 TG-25 leaves: wild-type vs. *lw1* edited.

Wild-type (A-C), *lw1* edited TG-25h (E-G) and TG-25c (I-K) leaf samples; one leaf sample was taken from each corresponding intact plant (D, H, L) for visualization and imaging under different magnifications. Asterisks identify same yellow/white stripe in each set of images; vertical lines in E-F and I-J span the widths of yellow/white stripes at the different magnifications. Confocal images (A, E, I), using a Zeiss LSM 510 confocal mounted on a Zeiss Axiovert 200 M inverted microscope, show autofluorescence (red) of chloroplasts. Bright field images, captured using a Keyance VHX7000 digital microscope, show magnified (B, F, J) and entire leaf width (C, G, K) of samples. Size bars: A, E, I = 50 µm, B, F, J = 200 µm.

A

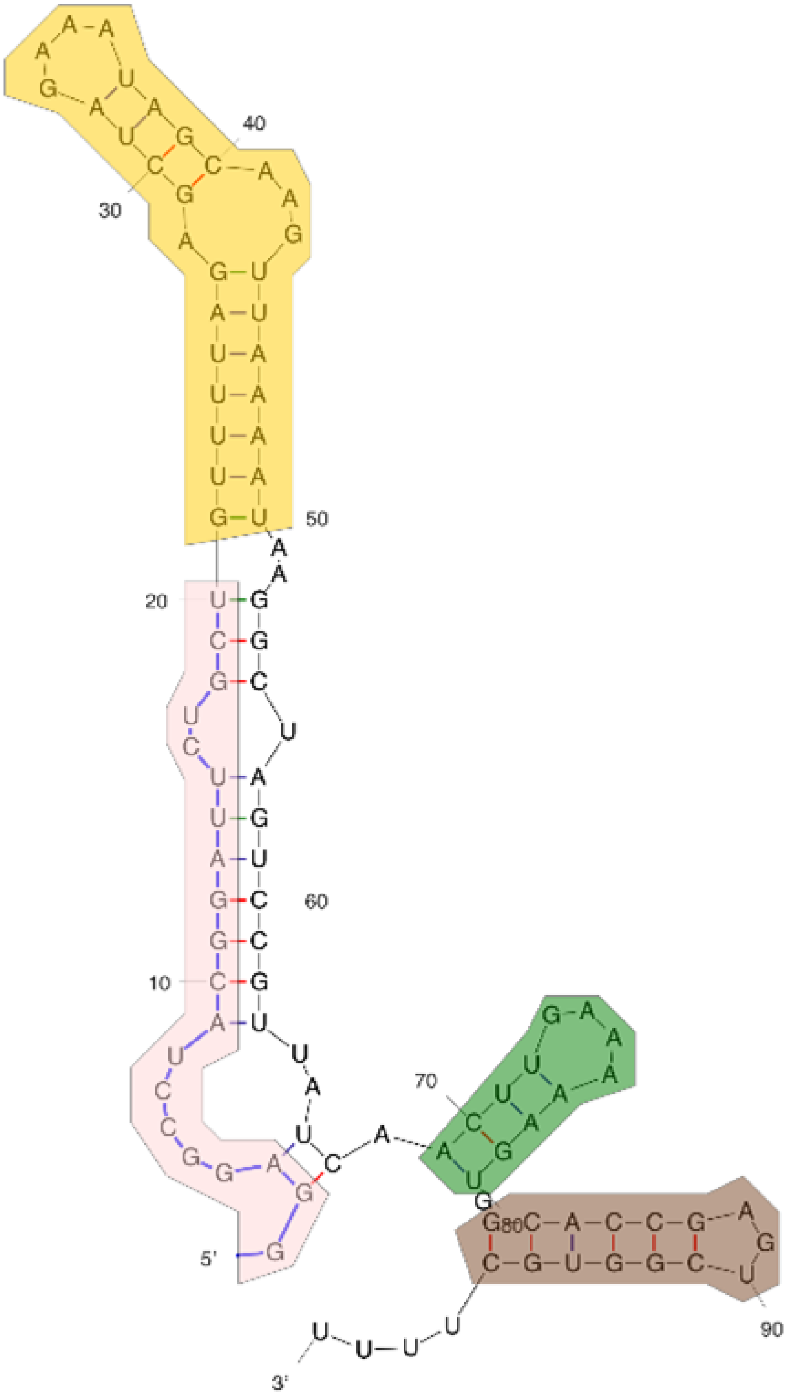

dG = -32.30 [Initially -31.20] lw1G1

B

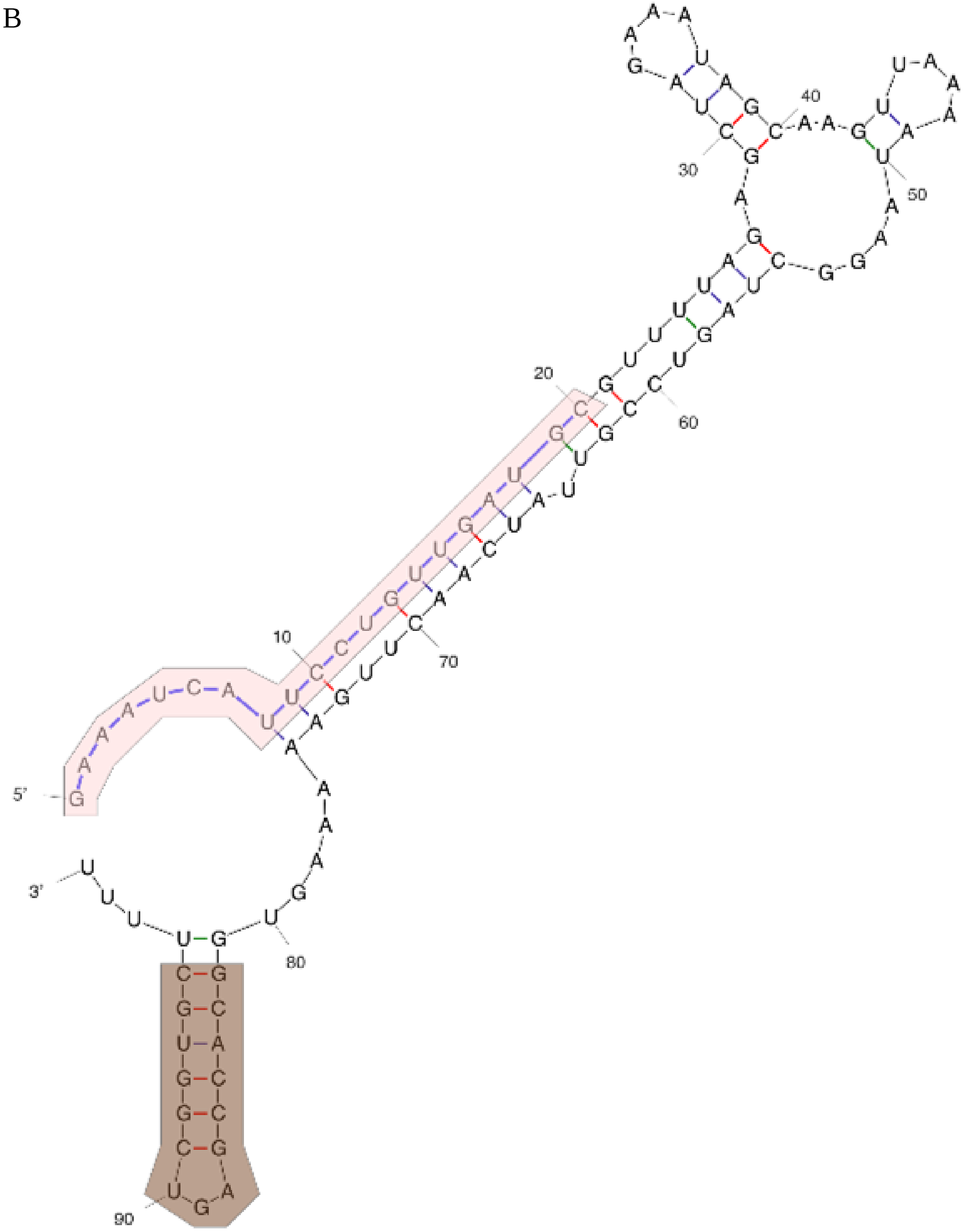

dG = -24.43 [Initially -27.30] lw1G2

C

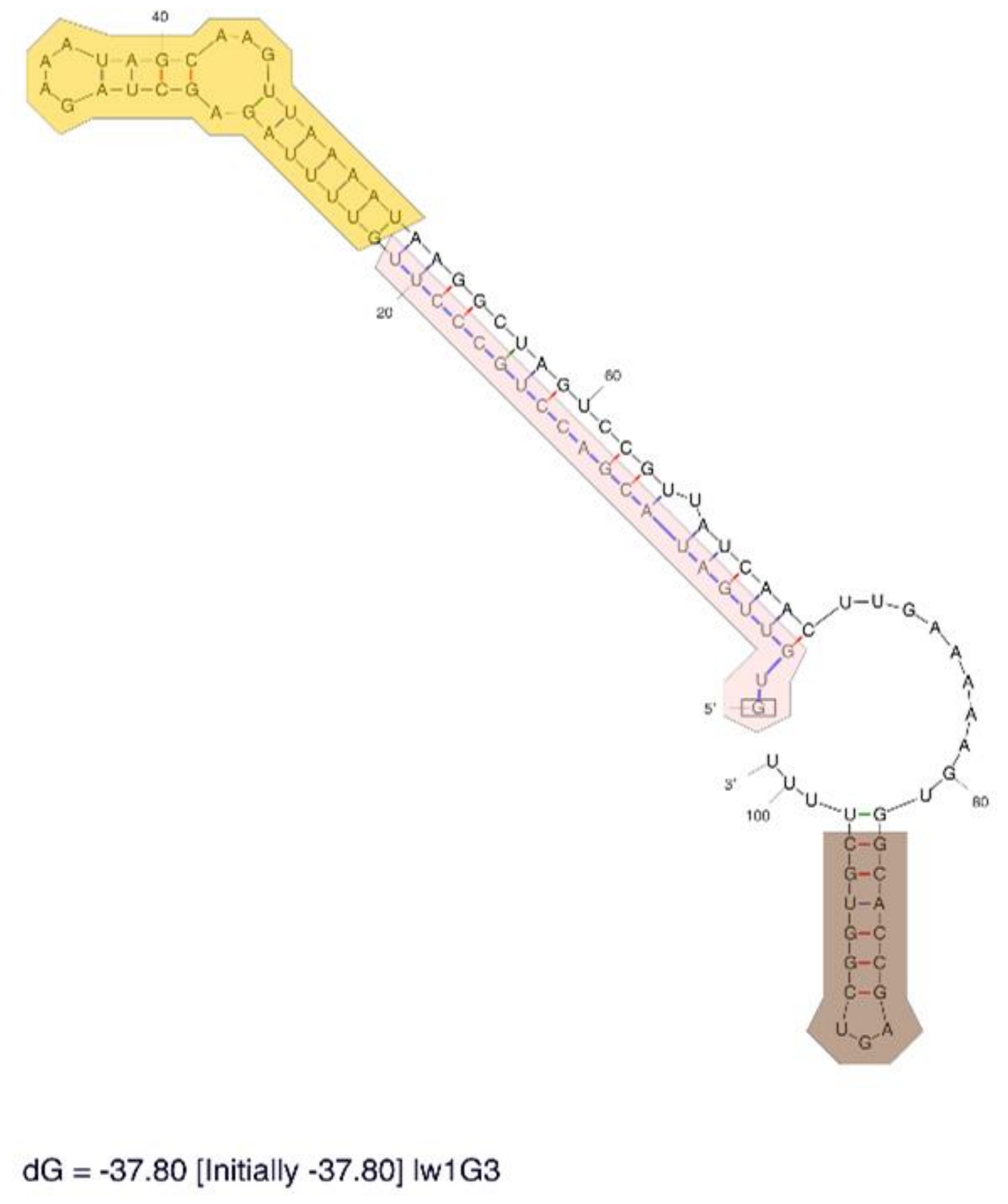

Fig. S7. Secondary structure prediction of three *lw1* single guide RNA (sgRNA) designs.
(A) lw1G1. (B) lw2G2. (C) lw1G3; nucleic acid base enclosed in box was added to the guide RNA to facilitate expression under U6 promoter. Guide RNA sequence is shaded in pink, repeat and anti-repeat stem loop is shaded in yellow, stem loop 2 is shaded in green, and stem-loop 3 is shaded in brown.
